# Speckle tracking derived reference values of myocardial deformation and impact of cardiovascular risk factors – Results from the population-based STAAB cohort study

**DOI:** 10.1371/journal.pone.0221888

**Published:** 2019-09-12

**Authors:** Caroline Morbach, Bettina N. Walter, Margret Breunig, Dan Liu, Theresa Tiffe, Martin Wagner, Götz Gelbrich, Peter U. Heuschmann, Stefan Störk, on behalf of the STAAB consortium

**Affiliations:** 1 Comprehensive Heart Failure Center, University and University Hospital Würzburg, Würzburg, Germany; 2 Department of Medicine I, Cardiology, University Hospital Würzburg, Würzburg, Germany; 3 Institute of Clinical Epidemiology and Biometry, University of Würzburg, Würzburg, Germany; 4 Clinical Trial Center, University Hospital Würzburg, Würzburg, Germany; Scuola Superiore Sant'Anna, ITALY

## Abstract

**Aims:**

We aimed to provide reference values for speckle-tracking derived systolic and diastolic myocardial deformation markers, and to determine their relation with age, sex, and cardiovascular risk factors.

**Methods and results:**

The *Characteristics and Course of Heart Failure STAges A/B and Determinants of Progression* (STAAB) cohort study recruited a representative sample of the population of Würzburg, Germany, aged 30–79 years. In a sample of 1818 participants (52% female, mean age 54±12 years) global longitudinal peak systolic strain (GL-PSS, n = 1218), systolic (GL-SSR, n = 1506), and early (GL-EDSR, n = 1506) and late diastolic strain rates (GL-LDSR, n = 1500) were derived from 2D speckle tracking analysis. From a subgroup of 323 individuals without any cardiovascular risk factor, sex- and age-specific reference values were computed. GL-PSS, GL-SSR, and GL-EDSR were associated with sex, GL-EDSR decreased and GL-LDSR increased with age. In the total sample, dyslipidemia was associated with altered GL-PSS, GL-SSR, and GL-EDSR in women but not in men, whereas obesity was associated with less favorable GL-PSS and GL-EDSR in either sex. Hypertension impacted more adversely on systolic and diastolic myocardial deformation in women compared to men (all p<0.01).

**Conclusion:**

The female myocardium appeared more vulnerable to high blood pressure and dyslipidemia when compared to men, while obesity was associated with adverse myocardial deformation in either sex. The reference values for echocardiographic myocardial deformation provided for a non-diseased population and their here reported associations with cardiovascular risk factors will inform future observational and intervention studies regarding i) effect sizes and power calculation, ii) cross-study comparisons, and iii) categorization of myocardial deformation in specific patient groups.

## Introduction

Echocardiography is the most frequently used method in the assessment of cardiac function. Conventional measurements like left ventricular (LV) ejection fraction are of limited utility to detect changes over time, hence, more sensitive methods are required. Strain as a measure of myocardial deformation carries incremental information on the change of the LV shape during the cardiac cycle [1]. Strain imaging may detect subtle alterations in cardiac function [2]. Two-dimensional speckle-tracking assesses myocardial motion by tracking speckles in the ultrasonic image. This method determines strain and strain rates avoiding Doppler-associated angulation errors and tethering artifacts with a good correlation to sonomicrometry and tagged magnetic resonance imaging (r = 0.87) [3]. Typically, the impairment in longitudinal deformation precedes deterioration of radial and/or circumferential deformation [4, 5].

The ability to quantify abnormal function relies on the definition of “normal”. Longitudinal systolic strain has consistently been reported more negative in women compared to men [6–13] indicating the necessity to apply sex-specific normal values. In contrast, the association of systolic strain patterns with age are contradictory [6–14], and knowledge on the association of diastolic myocardial deformation with age and sex is scarce. Importantly, there are no reference values available for speckle-tracking derived diastolic strain rates.

The adjustment of LV function to physiologic ageing is heavily influenced by the presence and individual expression of cardiovascular (CV) risk factors [15]. However, knowledge on their age-modifying effect on systolic and/or diastolic myocardial deformation is scarce. These small-scaled studies predominantly investigated selected age groups and isolated risk factors [5, 16–28]

We therefore aimed a) to establish speckle tracking derived sex- and age-specific normal values for systolic and diastolic myocardial deformation from a carefully selected group of individuals in sinus rhythm free of CV risk factors, and b) to determine the impact of age, sex, and classical CV risk factors on myocardial deformation.

## Methods

### Study population and recruitment

This is a prospectively planned analysis of the *Characteristics and Course of Heart Failure Stages A-B and Determinants of Progression* (STAAB) Cohort Study, based on consecutive participants from the general population of Würzburg, Germany, enrolled up to December 31, 2015. The detailed study design and methodology has been published [29]. A brief description is given in the *supporting information*.

### Cardiovascular risk factors

Prevalence of diabetes mellitus, CV disease (previous myocardial infarction, coronary artery disease, stroke, peripheral artery disease), and current pharmacotherapy was assessed by physician-led face-to-face interview. Assessment of smoking status, height, weight, and blood pressure, and an oral glucose tolerance test were performed according to standard operating procedures by trained and certified personnel [29]. Fasting lipid profile and glycosylated hemoglobin (HbA1c) were measured at the central laboratory of the University Hospital Würzburg. CV risk factors were defined according to current recommendations as follows: hypertension = blood pressure ≥140/90 mmHg [30] or anti-hypertensive pharmacotherapy; dyslipidemia = low density lipoprotein ≥190 mg/dl [31] or lipid-lowering pharmacotherapy; obesity = body mass index >30 kg/m^2^ [32]; diabetes mellitus = HbA1c >6.5%, fasting plasma glucose >7.0 mmol/l or 2h-plasma glucose >11.1 mmol/l [33] or anti-diabetic medication; smoking = current or ex-smoker.

All individuals with valid assessment of myocardial deformation entered the analyses regarding the impact of CV risk factors on myocardial deformation. For determination of normal values, we defined a sub-sample of healthy individuals, i.e. subjects in sinus rhythm and free from CV risk factors or CV disease.

### Echocardiography

The characteristics and effectiveness of performance measures of the echocardiographic quality assurance program established for the STAAB cohort study have been published [29, 34]. Image acquisition was performed by trained and certified sonographers on one echocardiography machine (Vivid S6^®^, M4S Sector Array Transducer operating at 1.5–4.3 MHz, GE Healthcare, Horten, Norway) with consistent system presets according to a pre-specified protocol [29, 34]. A minimum of three cardiac cycles was recorded. Standard LV apical views were acquired avoiding LV foreshortening with a frame rate of 50 to 80s^-1^, thus compatible with speckle tracking analysis. For tissue Doppler imaging (TDI) based reference assessments of myocardial deformation, additionally, small-angled images with high frame rates (80–100 s^-1^) were collected from the LV septal and lateral walls.

LV myocardial deformation was assessed offline using Q-Analysis (EchoPAC^®^ PC Version 113, GE Healthcare, Buckinghamshire, Great Britain). Timing of aortic valve closure was determined using continuous-wave Doppler across the aortic valve. Systolic as well as early and late diastolic SR at the time of peak S, peak E and peak A, respectively, were measured in each apical view and averaged to generate global longitudinal systolic (GL-SSR) as well as early diastolic (GL-EDSR) and late diastolic SR (GL-LDSR). Global longitudinal peak systolic strain (GL-PSS) was automatically averaged from individually calculated segmental strain values. If more than two out of 18 LV segments were insufficiently tracked, the individual was excluded from GL-PSS analysis. Nevertheless, all LV segments that could be analyzed entered segment-specific analyses. For variability assessment and in accordance with standard operating procedures of the quality control program [29, 34], 10 recordings were interpreted by two observers and by one observer twice, 10–14 days apart, blinded to the previous results. For validation of speckle tracking versus TDI based strain imaging, TDI based GL-EDSR of the LV mid-septum and mid-lateral wall was determined in 25 random subjects (detailed description provided in *supporting information*).

### Data analysis

Statistical analysis was performed using SPSS (Version 23, SPSS Inc., Chicago, USA). Descriptives of quantitative data are provided as mean and standard deviation. The relationship of global strain and SR with age and risk factors was examined by analysis of covariance. Main and interaction effects of CV risk factors on GL-PSS and SR were assessed using a general linear model. Age and sex were defined as main effects for analyses in the healthy sub-sample, and “no CV risk factor” plus individual CV risk factor for analyses in the total sample, respectively. P-values <0.05 were considered statistically significant. Observer variability was assessed using Bland-Altman 95% limits of agreement.

## Results

**Fig 1:**
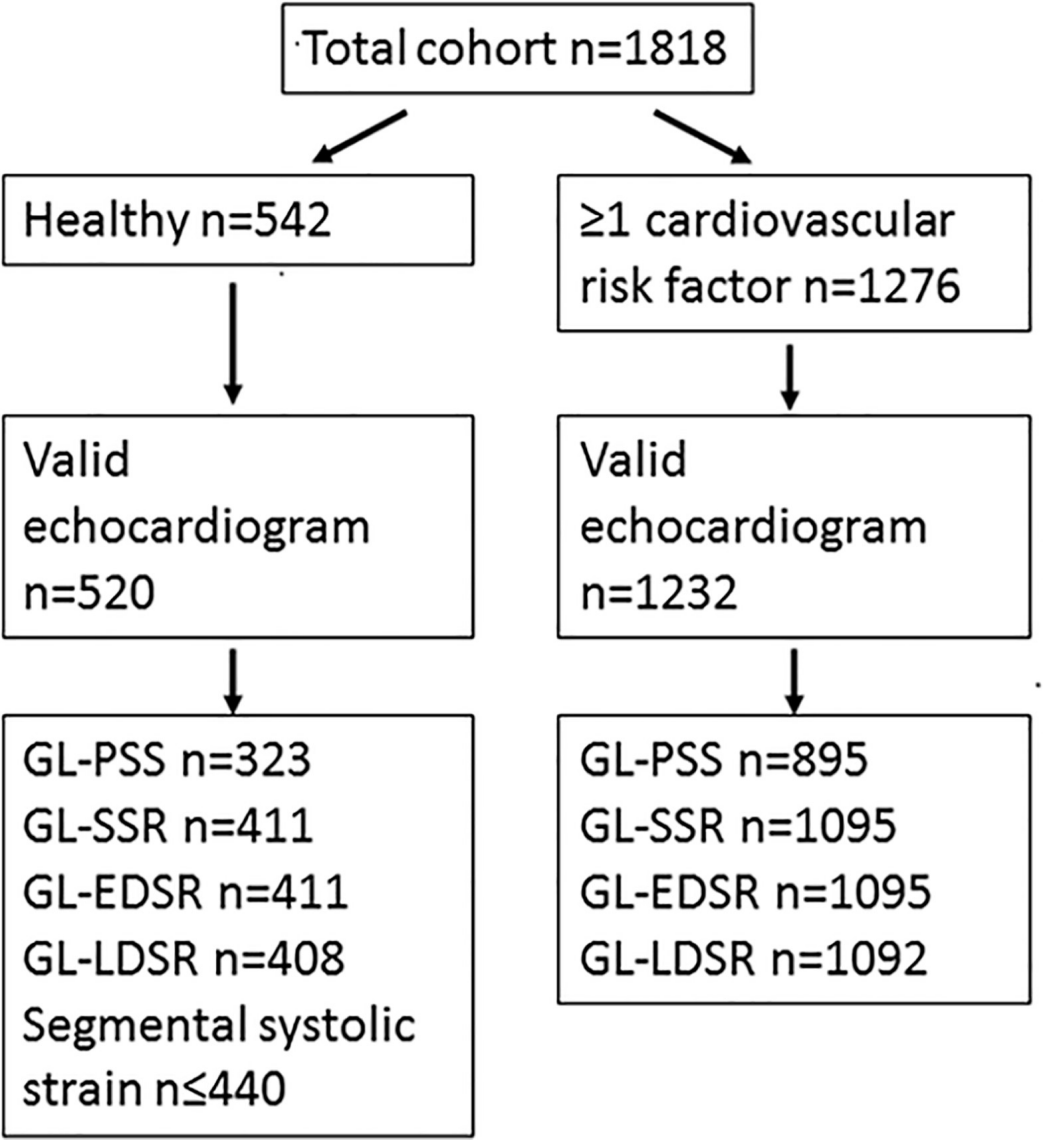
Number of individuals in each sub-sample in whom strain parameters could be derived. GL-PSS = global longitudinal peak systolic strain, GL-SSR = global longitudinal systolic strain rate, GL-EDSR = global longitudinal early diastolic strain rate, and GL-LDSR = global longitudinal late diastolic strain rate.

In the frame of the first planned interim analysis, we analyzed 1818 STAAB participants (mean age 54±12 years, 51.5% women). Of those, 542 (30%) participants qualified for the sub-sample of healthy individuals (49±11 years, 58% women) and 1276 exhibited at least one CV risk factor (Table 1, Fig 1).

**Table 1:** Clinical and echocardiographic characteristics of study participants, and subgroups without and with cardiovascular risk factors.

	Total	Healthy	With cardiovascular risk factor	P
n	1818	542	1276	
Age [years], mean (SD)	54 (12)	49 (11)	56 (12)	<0.001
Female sex, n (%)	937 (52)	313 (58)	624 (49)	<0.001
BMI [kg/m^2^], mean (SD)	26 (9)	24 (3)	28 (11)	<0.001
Systolic BP [mmHg], mean (SD)	124 (15)	118 (11)	127 (16)	<0.001
Diastolic BP [mmHg], mean (SD)	75 (10)	72 (9)	76 10)	<0.001
Heart rate [min^-1^], mean (SD)	62 (13)	62 (9)	63 (15)	0.929
Total cholesterol [mg/dl], mean (SD)	20 (38)	202 (35)	208 (39)	0.001
HDL cholesterol [mg/dl], mean (SD)	64 (19)	68 (18)	62 (19)	<0.001
LDL cholesterol [mg/dl], mean (SD)	121 (35)	117 (31)	123 (36)	<0.001
Triglycerides [mg/dl], mean (SD)	108 (81)	84 (43)	119 (91)	<0.001
HbA1c [%], mean (SD)	5.5 (0.6)	5.3 (0.3)	5.6 (0.6)	<0.001
**Echocardiography**				
**Frame Rate [s**^**-1**^**]**				
N	1752	520	1232	
mean (SD)	53 (11)	53 (10)	53 (11)	0.387
**GL-PSS [%]**				
N	1218	323	895	
mean (SD)	-19.1 (2.4)	-19.7 (2.2)	-18.9 (2.5)	<0.001
**Systolic SR [s**^**-1**^**]**				
N	1506	411	1095	
mean (SD)	-0.95 (0.15)	-0.98 (0.14)	-0.94 (0.15)	<0.001
**Early diastolic SR [s**^**-1**^**]**				
N	1506	411	1095	
mean (SD)	1.19 (0.37)	1.32 (0.36)	1.13 (0.36)	<0.001
**Late diastolic SR [s**^**-1**^**]**				
N	1500	408	1092	
mean (SD)	0.80 (0.21)	0.82 (0.21)	0.76 (0.19)	<0.001
**LVEDD [mm]**				
N	1752	520	1232	
mean (SD)	48.4 (4.7)	47.6 (4.5)	48.8 (4.8)	<0.001
**IVSd [mm]**				
N	1711	482	1229	
mean (SD)	8.7 (1.3)	8.1 (1.1)	8.9 (1.3)	<0.001
**LVPWd [mm]**				
N	1710	482	1228	
mean (SD)	8.2 (1.2)	7.5 (1.1)	8.4 (1.2)	<0.001
**LA area [cm**^**2**^**]**				
N	1689	499	1190	
mean (SD)	16.8 (3.1)	15.7 (2.7)	17.2 (3.1)	<0.001
**LVEF [%]**				
N	1729	517	1212	
mean (SD)	60.4 (4.5)	61.0 (4.1)	60.1 (4.6)	<0.001
**e´ [m/s]**				
N	1720	516	1204	
mean (SD)	0.11 (0.03)	0.12 (0.03)	0.10 (0.03)	<0.001
**E/e´**				
N	1714	515	1199	
mean (SD)	7.0 (2.3)	6.3 (1.7)	7.3 (2.5)	<0.001

Values are given as mean ± standard deviation. P values refer to the comparison of healthy individuals versus individuals with cardiovascular risk factors.

BP = blood pressure, HDL = high density lipoprotein, LDL = low density lipoprotein, HbA1c = hemoglobin A1c, GL-PSS = global longitudinal peak systolic strain, SR = strain rate, LVEDD = left ventricular end-diastolic diameter, IVSd = interventricular septum end-diastolic, LVPWd = left ventricular posterior wall end-diastolic, LA = left atrium, LVEF = left ventricular ejection fraction, e´ = PW-Doppler derived early diastolic myocardial lengthening velocity, E = early mitral inflow velocity

Owing to the preselection on risk, participants with CV risk factors featured numerous differences compared to the healthy group: they were older, had higher body mass index, blood pressure, cholesterol values, and also a higher HbA1c (Table 1). Accordingly, most echocardiographic markers matched with this adverse profile. Participants with CV risk factors had lower values for LVEF, GL-PSS, and all types of SR, but higher values for E/e´, LV end-diastolic diameter, septal and posterior wall thickness, and left atrial size. Of note, heart rate and frame rate of echocardiographic image acquisition were similar between groups (Table 1).

Although the distribution of sex was balanced across the five age categories in the total sample (p = 0.41), subjects with CV risk factors were expectedly older than healthy subjects.

In a total of 1752 individuals with valid echocardiograms, feasibility was 70% for GL-PSS and 86% for strain rates, respectively. Age, body mass index, heart rate, and frame rate had no impact on feasibility to derive GL-PSS measurement, but individuals with valid GL-PSS were significantly more often male (624 men vs. 594 women, p = 0.01). The feasibility to derive any modality of SR was significantly associated with younger age, male sex, and lower body mass index (all p<0.05).

For GL-PSS, GL-SSR, GL-EDSR, and GL-LDSR, the 90^th^ percentiles of the absolute difference of two interpretations were 0.8%, 0.05 s^-1^, 0.08 s^-1^, and 0.04 s^-1^ for repeated interpretation by the same observer, and 2.6%, 0.16 s^-1^, 0.01 s^-1^, and 0.03 s^-1^ for the interpretation by two observers, respectively. GL-EDSR derived by speckle tracking and TDI was 1.40±0.68 s^-1^ and 1.89±0.56 s^-1^, respectively; the correlation coefficient for both methods was r = 0.70 [95%CI 0.59–0.80] (*Figure A in S1 File*).

### Normal values for myocardial deformation in individuals free from CV risk factors and CV disease

#### Systolic strain

**Fig 2:**
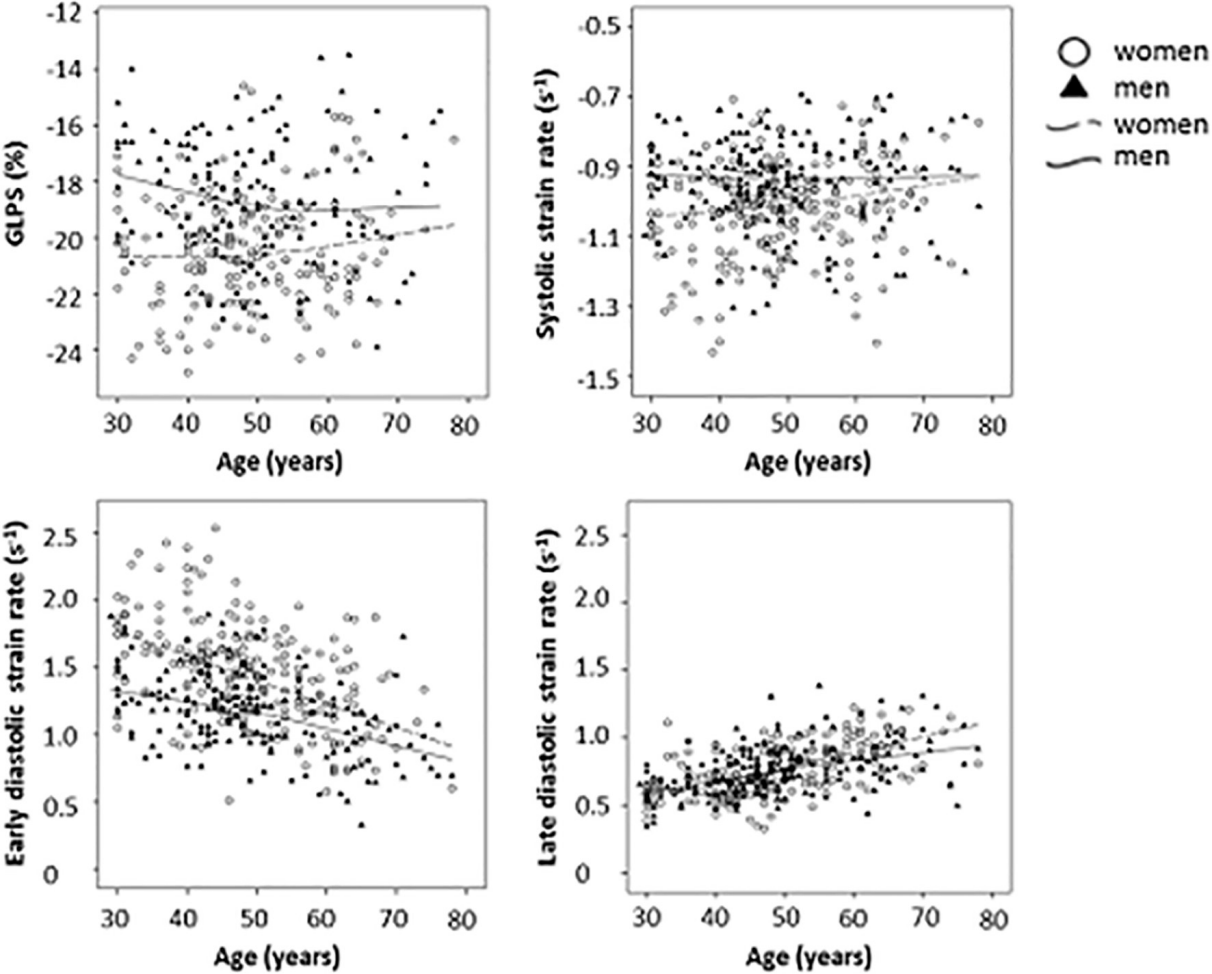
Combined averaged a) global longitudinal peak systolic strain (GL-PSS, n = 323), b) systolic strain rate (GL-SSR, n = 410), c) early diastolic strain rate (GL-EDSR, n = 410), and d) late diastolic strain rate (GL-LDSR, n = 407) according to age and sex in individuals in sinus rhythm free from cardiovascular risk factors and cardiovascular disease (mean age 49±11 years, 55% females).

GL-PSS values could be assessed in 323 healthy individuals and were normally distributed (*Figure B in S1 File*). In a linear model, there was a non-significant change of GL-PSS of -0.23% per age decade in men (p = 0.131) and of +0.29% per age decade in women (p = 0.054); however, the slopes for both sexes differed significantly (p = 0.015; Fig 2). Overall, regardless of age, GL-PSS was by 1.74% more negative in women compared to men (p<0.001). Sex-specific normal values per age decades are given in Tables 2 and 3.

**Table 2:** Speckle tracking derived markers of left ventricular myocardial deformation in male participants by age group.

Age group (years)	30–39	40–49	50–59	60–79	P
**GL-PSS [%]**					
**N**	25	53	38	30	
**mean (SD)**	-17.8 (1.7)	-18.9 (2.0)	-19.0 (2.3)	-18.8 (2.5)	0.160
**2SD range**	-21.3; -14.4	-22.9; -14.9	-23.6; -14.4	-23.8; -13.8	
**Systolic strain rate [s^-1^]**					
**N**	34	70	39	40	
**mean (SD)**	-0.93 (0.10)	-0.95 (0.13)	-0.95 (0.18)	-0.93 (0.14)	0.691
**2SD range**	-1.13; -0.73	-1.21; -0.69	-1.31; -0.59	-1.21; -0.65	
**Early diastolic strain rate [s^-1^]**					
**N**	34	70	39	40	
**mean (SD)**	1.30 (0.26)	1.23 (0.24)	1.16 (0.28)	0.95 (0.27)	<0.001
**2SD range**	0.78; 1.82	0.75; 1.71	0.60; 1.72	0.41; 1.49	
**Late diastolic strain rate [s^-1^]**					
**N**	34	69	39	40	
**mean (SD)**	0.62 (0.13)	0.75 (0.18)	0.82 (0.17)	0.87 (0.21)	<0.001*
**2SD range**	0.36; 0.88	0.39; 1.11	0.48; 1.16	0.45; 1.29	

Values are given as mean ± standard deviation. P values refer to the comparison of age groups using ANCOVA or *Welch´s test, depending on equality of variance in Levene´s test. To increase statistical power, the two top decades were combined.

SD = standard deviation, CI = confidence interval, GL-PSS = global longitudinal peak systolic strain

**Table 3:** Speckle tracking derived markers of left ventricular myocardial deformation in female participants by age group.

Age group (years)	30–39	40–49	50–59	60–79	P
**GL-PSS [%]**					
**N**	28	73	42	34	
**mean (SD)**	-20.7 (2.2)	-20.5 (1.8)	-20.7 (1.6)	-19.6 (2.4)	0.120*
**2SD range**	-25.1; -16.3	-24.1; -16.9	-24.1; -17.3	-24.4; -14.8	
**Systolic strain rate [s^-1^]**					
**N**	38	97	53	40	
**mean (SD)**	-1.06 (0.15)	-1.02 (0.13)	-1.02 (0.10)	-0.98 (0.15)	0.174*
**2SD range**	-1.36; -0.76	-1.28; -0.76	-1.22; -0.82	-1.28; -0.68	
**Early diastolic strain rate [s^-1^]**					
**N**	38	97	53	40	
**mean (SD)**	1.69 (0.35)	1.52 (0.35)	1.35 (0.27)	1.15 (0.36)	<0.001
**2SD range**	0.99; 2.39	0.82; 2.22	0.81; 1.89	0.43; 1.87	
**Late diastolic strain rate [s^-1^]**					
**N**	37	97	52	40	
**mean (SD)**	0.65 (0.15)	0.69 (0.15)	0.82 (0.15)	0.94 (0.16)	<0.001
**2SD range**	0.35; 0.95	0.39; 0.99	0.52; 1.12	0.62; 1.26	

Values are given as mean ± standard deviation. P values refer to the comparison of age groups using ANCOVA or *Welch´s test, depending on equality of variance in Levene´s test. To increase statistical power, the two top decades were combined. SD = standard deviation, CI = confidence interval, GL-PSS = global longitudinal peak systolic strain

We provide sex-specific percentiles for GL-PSS (*Figures C and D in S1 File*) as well as age- and sex specific systolic strain values for each left ventricular segment (*Tables A and B in S1 File*). In women, basal septal, mid septal, basal inferior, as well as all anteroseptal segments showed a significantly less negative strain with increasing age, whereas in men systolic strain remained unchanged in all segments.

#### Strain rate

Sex-specific normal values per age decades are given in Tables 2 and 3. GL-SSR changed by -0.003 s^-1^ per age decade in men (p = 0.741), and +0.023 s^-1^ per decade in women (p = 0.007), with a significant difference between slopes (p = 0.032). Regardless of age, women had 0.072 s^-1^ more negative values compared to men (p<0.001; Fig 2).

GL-EDSR changed by -0.106 s^-1^ per age decade in men (p<0.001), and -0.175 s^-1^ per age decade in women (p<0.001), with a significant difference between slopes (p = 0.011). Regardless of age, women had 0.275/s^-1^ more positive values compared to men (p<0.001).

GL-LDSR changed by +0.074 s^-1^ per age decade in men (p<0.001), and +0.100 s^-1^ per age decade in women (p<0.001,) without a significant difference between slopes (p = 0.080). Regardless of age, women had 0.006 s^-1^ less positive values compared to men (p = 0.747).

Sex- and age-specific percentiles of GL-SSR, GL-EDSR, and GL-LDSR are detailed in *Figures E-J in S1 File.*

### Impact of CV risk factors on myocardial deformation

1276 individuals exhibited at least one CV risk factor (56±12 years, 49% women). The number of individuals decreased with increasing number of prevalent CV risk factors and was evenly distributed over the decades (*Figures K and L in S1 File*).

In the total sample, GL-PSS was adversely affected by obesity in either sex (p<0.001), whereas an adverse effect of hypertension and dyslipidemia on GL-PSS was selectively observed in women (Table 4, *Table C in S1 File*). An adverse effect of dyslipidemia, hypertension, and obesity on GL-SSR was consistently observed in women only (Table 4, *Table D in S1 File*). GL-EDSR was negatively affected by hypertension and dyslipidemia, with a significantly more adverse effect in women, and by obesity in either sex (Table 4, *Table E in S1 File*). GL-LDSR was significantly increased in individuals with hypertension in either sex, with a significantly more adverse effect in women (Table 3, *Table F in S1 File*). Diabetes mellitus and smoking had no significant adverse effect on myocardial deformation (*Tables C-F in S1 File*)

**Table 4:** Impact of cardiovascular risk factors on myocardial deformation in the total cohort and according to sex.

	CV risk factor	Impact of CV risk factor	*P*	Effect size women vs. men	*P* for effect in women	*P* for effect in men	*P* for interaction
**GL-PSS**	Obesity	+0.7%	<0.001	+0.9% *vs*. +0.7%	<0.01	<0.01	0.69
	Hypertension	+0.3%	ns	+0.7% *vs*. -0.1%	<0.01	ns	0.004
	Dyslipidemia	+0.3%	<0.001	+1.2% *vs*. +0.3%	<0.001	ns	0.03
**GL-SSR**	Obesity	+0.04 s^-1^	<0.001	+0.06 s^-1^ *vs*. +0.01 s^-1^	<0.001	ns	0.047
	Hypertension	+0.03 s^-1^	<0.01	+0.06 s^-1^ *vs*. +0.02 s^-1^	<0.001	ns	0.02
	Dyslipidemia	+0.03 s^-1^	<0.05	+0.05 s^-1^ *vs*. +0.01 s^-1^	<0.01	ns	0.07
**GL-EDSR**	Obesity	-0.12 s^-1^	<0.001	-0.14 s^-1^ *vs*. -0.12 s^-1^	<0.001	<0.001	0.72
	Hypertension	-0.72 s^-1^	<0.001	-0.24 s^-1^ *vs*. -0.10 s^-1^	<0.001	<0.001	<0.001
	Dyslipidemia	-0.12 s^-1^	<0.001	-0.19 s^-1^ *vs*. -0.2 s^-1^	<0.001	ns	0.001
**GL-LDSR**	Hypertension	+0.09 s^-1^	<0.001	+0.11 s^-1^ *vs*. +0.06 s^-1^	<0.001	<0.001	0.02

The impact of a CV risk factor on a specific strain marker is expressed as absolute change for the total sample and per sex group. Interaction effects computed from general linear models (see Methods).

CV = cardiovascular, vs. = versus, GL-PSS = global longitudinal peak systolic strain (n = 1218), GL-SSR = global longitudinal systolic strain rate (n = 1506), GL-EDSR = global longitudinal early diastolic strain rate (n = 1506), GL-LDSR = global longitudinal late diastolic strain rate (n = 1500), ns = not significant; Hypertension = blood pressure ≥140/90 mmHg or antihypertensive pharmacotherapy, dyslipidemia = low density lipoprotein ≥190 mg/dl or lipid-lowering pharmacotherapy, obesity = body mass index >30 kg/m^2^.

## Discussion

From a well-characterized, population-based cohort balanced for age and sex, we defined a sub-sample of healthy individuals (in sinus rhythm and free of CV risk factors and CV disease) and established reference values for global and segmental peak systolic strain and systolic SR of the LV. To the best of our knowledge, the current report is first to provide speckle-tracking derived reference values for early and late diastolic SR. Systolic and early, but not late, diastolic myocardial deformation showed a strong association with sex. Additionally, in contrast to systolic deformation parameters, diastolic SR markers were strongly affected by age: GL-EDSR decreased, while GL-LDSR increased with age.

In the total sample, CV risk factors differentially affected the various aspects of myocardial deformation. Further, sex-specific effects of CV risk factors on myocardial deformation were observed. This is compatible with the hypothesis that the myocardial sensitivity to individual risk factors is determined by sex.

### Quality assurance

Assessment of acquisition variability and interpretation variability confirmed sound agreement between observers. Applying high quality standards to image and tracking quality, feasibility of GL-PSS in the total sample was 70%, which is comparable to other larger studies [11, 12]. Compared to TDI, speckle tracking derived GL-EDSR, which due to the highest velocity is most prone to undersampling by lower frame rates, indeed yielded a systematic deviation exhibiting slightly lower values (factor 0.7). Nevertheless, the good correlation between both methods and comparable standard deviation (1.40±0.68 s^-1^ versus 1.89±0.56 s^-1^) justify the clinical application of speckle tracking derived GL-EDSR. These findings emphasize the need for population-based normal values specifically derived from speckle tracking based strain imaging.

### Systolic myocardial deformation in healthy individuals

In healthy individuals, GL-PSS and SR were found more negative in women compared to men [11], with disparate results regarding their association with age [11]. As age advances, the relationship of cardiac structure and function with age is confounded by the accumulation of traditional risk factors [15]. Most echocardiographic studies describing an association of GL-PSS with age did not systematically exclude individuals with CV risk factors or overt CV disease [11, 35, 36], which is the likely reason for these incongruent results.

We performed a detailed, physician-based assessment and thus established a well selected sub-sample of “truly healthy” individuals, i.e. in sinus rhythm and free from CV risk factors and CV disease. We here confirmed a more negative GL-PSS in women compared to men. Further, we found no significant change of systolic myocardial deformation with age in men, but significantly less positive GL-SSR with advancing age and a trend towards less negative GL-PSS accompanied by a significant impairment of segmental GL-PSS in septal and anteroseptal segments in women. This is in line with results from the EACVI NORRE study, where the pattern of worse systolic longitudinal LV function with advancing age in women was associated with more negative values of circumferential strain [37]. More detailed assessment of the underlying pathomechanisms including hormonal analyses and the evaluation of other than the conventional cardiovascular risk factors will be subjected to further research.

### Diastolic myocardial deformation in healthy individuals

GL-EDSR is considered a comprehensive measure of early active LV relaxation. Importantly, diastolic SR yielded higher accuracy regarding the estimation of LV filling pressures compared to indices including the broadly used but angle dependent and mono-dimensional TDI measurement e´ [38, 39]. Further, GL-LDSR is considered a measure of late diastolic LV filling induced by active atrial contraction. Our analyses extend previous knowledge, as we found that GL-EDSR was significantly higher in women compared to men, whereas no sex-related difference was apparent regarding GL-LDSR. Further, GL-EDSR significantly decreased, whereas GL-LDSR significantly increased with age. This implies an increase of active atrial contribution to LV filling with advancing age, thus possibly compensating for the described decrease in active LV relaxation.

### Impact of CV risk factors on systolic and diastolic myocardial deformation

A recent report from the MESA study employing cardiac magnet resonance tomography reported that CV risk status–besides sex and ethnicity–were major drivers of the progression of LV measures [15]. Using echocardiography, hypertensive heart disease with normal ejection fraction has been associated with reduced myocardial velocities and reduced regional function [40], and diabetes mellitus with worse LV remodeling and function [26, 28]. Results regarding the impact of obesity on myocardial function are inconsistent, reporting negative, positive or neutral associations of LV diastolic function patterns with the degree of obesity [5, 20, 22, 24, 25, 27, 41]. Two larger studies reported neutral findings [24, 25] and emphasized the importance of factors defining the metabolic syndrome rather than obesity itself. According to one report comparing 40 otherwise healthy smokers with age-matched controls, smoking intensity gradually impaired systolic and diastolic myocardial deformation patterns of both the left and the right ventricle [23]. Hence, evidence of the negative impact of CV risk factors on myocardial deformation is inconsistent, mainly due to heterogeneous study quality and relatively small samples looking at restricted age ranges and risk profiles.

To our knowledge, the current study is first to assess the individual impact of each of the established CV risk factors on systolic and diastolic myocardial deformation in a well-controlled representative cohort. Our results suggest a sex-specific sensitivity of the myocardium to individual CV risk factors. The vulnerability of the female myocardium to high blood pressure with subsequent alteration of the active early diastolic myocardial relaxation, for example, might be an explanation for the preponderance of females in HF with preserved ejection fraction. We did not observe any direct negative impact of smoking and diabetes mellitus on myocardial deformation at rest. These risk factors might act as dormant harmful factors affecting the vasculature, i.e. not affecting the myocardial function at rest until an ischemic damage has occurred. Further, all individuals with diabetes mellitus also exhibited at least one additional CV risk factor, which might have had a stronger impact on longitudinal LV function than diabetes mellitus.

### Strengths and limitations

Strain imaging depends on optimal image quality. Hence, feasibility is lower compared to routine echocardiography. Nevertheless, applying high quality standards, we achieved a feasibility comparable to other large cohort studies [11, 36]. The current analysis omitted radial and circumferential deformation as we focused on longitudinal myocardial deformation, which is affected first along the pathophysiological cascade [4]. CV risk factors were assessed very carefully. Nevertheless, more detailed analyses including pharmacotherapy and quality of cardiovascular risk factor control were not performed due to the sample size.

## Clinical impact and conclusion

Healthy aging seems to be associated with a selective decrease in systolic function in women. By contrast, active LV relaxation decreases with advancing age in either sex, necessitating the left atrium to contribute increasingly more to left ventricular filling. Further, the female myocardium appears more vulnerable to high blood pressure and dyslipidemia when compared to men, while obesity might reduce myocardial deformation to a similar extent in either sex.

The here presented sex- and age-specific speckle-tracking derived reference values for systolic and, importantly, also for diastolic myocardial deformation, will help to classify myocardial deformation in patients more reliably. Reference values of strain and strain rates and their here reported association with CV risk factors will inform future observational and intervention studies regarding i) effect sizes and power calculation, ii) cross-study comparisons, and iii) categorization of myocardial deformation in specific patient groups.

## Supporting information

S1 File Supplementary methods: Study population and recruitment, myocardial deformation imaging–detailed, Tissue-Doppler derived strain rate imaging, and data analysis. Supplementary tables: Table A. Left ventricular global and segmental peak systolic longitudinal strain in males. Table B. Left ventricular global and segmental peak systolic longitudinal strain in females. Table C. Impact of cardiovascular risk factors on global longitudinal peak systolic strain in the total cohort and according to sex. Table D. Impact of cardiovascular risk factors on systolic strain rate in the total cohort and according to sex. Table E. Impact of cardiovascular risk factors on early diastolic strain rate in the total cohort and according to sex. Table F. Impact of cardiovascular risk factors on late diastolic strain rate in the total cohort and according to sex. Supplementary figures: Figure A. Correlation of speckle tracking and tissue Doppler imaging derived global early diastolic strain rate. Figure B. Distribution of GL-PSS values in individuals without CVRF. Figure C. Percentiles of global longitudinal peak systolic strain in men. Figure D. Percentiles of global longitudinal peak systolic strain in women. Figure E. Percentiles of systolic strain rate in men. Figure F. Percentiles of systolic strain rate in women. Figure G. Percentiles of early diastolic strain rate in men. Figure H. Percentiles of early diastolic strain rate in women. Figure I. Percentiles of late diastolic strain rate in men. Figure J. Percentiles of late diastolic strain rate in women. Figure K. Number of male individuals with 0–5 cardiovascular risk factors by age. Figure L. Number of female individuals with 0–5 cardiovascular risk factors by age.
(DOCX)

## Abbreviations

CV: Cardiovascular
GL-EDSR: Global longitudinal early diastolic strain rate
GL-LDSR: Global longitudinal late diastolic strain rate
GL-PSS: Global longitudinal peak systolic strain
GL-SSR: Global longitudinal systolic strain rate
LV: Left ventricle/ventricular
STAAB: Characteristics and Course of Heart Failure STAges A/B and Determinants of Progression cohort study
SR: Strain rate
TDI: Tissue Doppler Imaging
