# Supplementary material for: Speckle tracking derived reference values of myocardial deformation and impact of cardiovascular risk factors – Results from the population-based STAAB cohort study
Source: PLoS One. 2019 Sep 12;14(9):e0221888. doi: 10.1371/journal.pone.0221888 (PMC6742365; doi:10.1371/journal.pone.0221888)
Supplement: S1 File — Supplementary tables: Table A. Left ventricular global and segmental peak systolic longitudinal strain in males. Table B. Left ventricular global and segmental peak systolic longitudinal strain in females. Table C. Impact of cardiovascular risk factors on global longitudinal peak systolic strain in the total cohort and according to sex. Table D. Impact of cardiovascular risk factors on systolic strain rate in the total cohort and according to sex. Table E. Impact of cardiovascular risk factors on early diastolic strain rate in the total cohort and according to sex. Table F. Impact of cardiovascular risk factors on late diastolic strain rate in the total cohort and according to sex. Supplementary figures: Figure A. Correlation of speckle tracking and tissue Doppler imaging derived global early diastolic strain rate. Figure B. Distribution of GL-PSS values in individuals without CVRF. Figure C. Percentiles of global longitudinal peak systolic strain in men. Figure D. Percentiles of global longitudinal peak systolic strain in women. Figure E. Percentiles of systolic strain rate in men. Figure F. Percentiles of systolic strain rate in women. Figure G. Percentiles of early diastolic strain rate in men. Figure H. Percentiles of early diastolic strain rate in women. Figure I. Percentiles of late diastolic strain rate in men. Figure J. Percentiles of late diastolic strain rate in women. Figure K. Number of male individuals with 0–5 cardiovascular risk factors by age. Figure L. Number of female individuals with 0–5 cardiovascular risk factors by age. (DOCX) [file pone.0221888.s001.docx]

**Speckle tracking derived reference values of myocardial deformation and impact of cardiovascular risk factors – Results from the population-based STAAB cohort study**

Morbach C, Walter B, Breunig M, Liu D, Tiffe T, Wagner M, Gelbrich G, Heuschmann P, Störk S

**On behalf of the STAAB consortium**: S. Frantz (Dept. of Medicine I, Div. of Cardiology, University Hospital Würzburg); C. Maack (Comprehensive Heart Failure Center, University Hospital and University of Würzburg); G. Ertl (University Hospital Würzburg); M. Fassnacht (Dept. of Medicine I, Div. of Endocrinology, University Hospital Würzburg); C. Wanner (Dept. of Medicine I, Div. of Nephrology, University Hospital Würzburg); R. Leyh (Dept. of Cardiovascular Surgery, University Hospital Würzburg); J. Volkmann (Dept. of Neurology, University Hospital Würzburg); J. Deckert (Dept. of Psychiatry, Psychosomatics and Psychotherapy, Center of Mental Health, University Hospital Würzburg); H. Faller (Dept. of Medical Psychology, University of Würzburg); R. Jahns (Interdisciplinary Bank of Biomaterials and Data Würzburg, University Hospital Würzburg).

**Supporting Information**

This appendix is provided by the authors to give readers additional information about their work.

Supplementary methods

Study population and recruitment

STAAB aims to determine the prevalence and natural course of the early stages of heart failure (HF) in a representative sample (n=5000) of the general population of Würzburg, Germany, aged 30-79 years. Sole exclusion criterion is the prevalence of symptomatic heart failure. The STAAB cohort study protocol and procedures received positive votes from the Ethics Committee of the Medical Faculty (vote 98/13) as well as from the data protection officer of the University of Würzburg (J-117.605-09/13). All participants provided written informed consent prior to any study examination.

Myocardial deformation imaging – detailed

Speckle-tracking derived strain and strain rate imaging

The timing of aortic valve closure was determined using continuous-wave Doppler across the aortic valve. Myocardial deformation imaging was performed offline using Q-Analysis (EchoPAC® PC Version 113, GE Healthcare, Buckinghamshire, Great Britain). A region of interest was created by manually outlining the endocardial border at end-systolic frame on the apical 4-chamber, 2-chamber and 3-chamber views. The system automatically tracked the tissue within the region of interest and divided the myocardium into six segments in each apical view, accounting for a total of 18 segments covering the entire LV from base to apex. The region of interest was manually adjusted for each segment if necessary to ensure optimal tracking quality. Systolic as well as early and late diastolic SR at the time of peak S, peak E and peak A, respectively, were measured in each apical view and averaged to generate global longitudinal systolic (GL-SSR) as well as early diastolic (GL-EDSR) and late diastolic SR (GL-LDSR). Global longitudinal peak systolic strain (GL-PSS) was automatically averaged from individually calculated segmental strain values. If more than two out of 18 LV segments were insufficiently tracked, the individual was excluded from GL-PSS analysis. Nevertheless, all LV segments that could be analyzed entered the segment-specific analyses.

Tissue-Doppler derived strain rate imaging

Real-time tissue-Doppler-imaging (TDI) loops with 3 consecutive cardiac cycles for the septal and lateral walls respectively were recorded from the standard apical 4-chamber view. TDI data were analyzed offline using dedicated software (EchoPAC^TM^ Software version 202, GE Ultrasound). A region of interest (ROI) was placed at mid segments of septal and lateral walls, respectively. The tilt angle of the sample area was parallel to both of the ventricular wall and the ultrasound beam. This ROI sample was continuously positioned within the wall during the cardiac cycle by manual tracking. Strain rates were derived by estimating the local spatial gradients in myocardial velocities. Strain rate curves were averaged over 3 cardiac cycles and integrated over time to derive natural strain curves using end-diastole as the reference point. Aortic valve opening and closure were extracted from conventional blood flow traces and were used to define the ejection period. TDI-derived early diastolic strain rate was measured in the mid-septum and mid-lateral wall.

Data analysis

TDI-derived early diastolic stain rates from the mid-septum and the mid-lateral wall were averaged. Using Pearson correlation and linear regression, this average value was compared to the speckle-tracking derived global early diastolic strain rate value of the respective individual.

Supplementary tables

**Table A:** Left ventricular global peak systolic longitudinal strain and – indented – the respective segmental longitudinal peak systolic strain values in male participants by age group

| Age group (years) | 30-39 | 40-49 | 50-59 | 60-79 | p |
| --- | --- | --- | --- | --- | --- |
|  |  |  |  |  |  |
| GL-PSS [%] |  |  |  |  |  |
| N | 25 | 53 | 38 | 30 |  |
| mean (SD) | -17.8 (1.7) | -18.9 (2.0) | -19.0 (2.3) | -18.8 (2.5) | 0.160 |
|  |  |  |  |  |  |
| LPS 4C [%] |  |  |  |  |  |
| N | 30 | 63 | 39 | 36 |  |
| mean (SD) | -17.2 (2.0) | -18.6 (2.3) | -18.5 (2.6) | -18.3 (2.6) | 0.052 |
| Basal septal [%]  N= 190  mean (SD) | 35  -15.4 (3.4) | 71  -15.0 (3.2) | 41  -15.6 (4.0) | 43  -14.3 (3.5) | 0.319 |
| Mid septal [%]  N= 190  mean (SD) | 35  -18.2 (2.9) | 71  -18.3 (2.5) | 41  -19.0 (2.7) | 43  -17.4 (5.8) | 0.263 |
| Apical septal [%]  N= 186  mean (SD) | 35  -20.5 (3.7) | 71  -22.2 (3.9) | 40  -22.1 (5.0) | 40  -22.2 (4.8) | 0.293 |
| Apical lateral [%]  N= 179  mean (SD) | 35  -18.2 (4.0) | 68  -18.8 (4.9) | 38  -18.9 (4.5) | 38  -18.4 (4.8) | 0.892 |
| Mid lateral [%]  N= 186  mean (SD) | 35  -17.0 (3.1) | 70  -18.0 (3.3) | 41  -16.9 (3.0) | -  40  17.1 (4.2) | 0.327 |
| Basal lateral [%]  N= 183  mean (SD) | 31  -16.8 (3.5) | 70  -18.0 (3.8) | 41  -18.0 (4.1) | 41  -20.8 (12.7) | 0.073 |
|  |  |  |  |  |  |
| LPS 2C [%]  N= 155  mean (SD) | 29  -19.0 (2.4) | 56  -19.4 (1.9) | 38  -19.5 (2.4) | 32  -19.6 (3.3) | 0.827* |
| Basal inferior [%]  N= 183  mean (SD) | 34  -19.4 (3.7) | 71  -18.9 (3.2) | 40  -20.0 (3.4) | 38  -18.9 (4.4) | 0.443 |
| Mid inferior [%]  N= 185  mean (SD) | 34  -20.0 (2.8) | 71  -19.4 (5.8) | 40  -21.0 (3.2) | 40  -20.3 (3.4) | 0.362 |
| Apical inferior [%]  N= 168  mean (SD) | 30  -21.0 (9.9) | 63  -22.1 (7.2) | 37  -22.6 (3.7) | 38  -23.5 (5.1) | 0.462 |
| Apical anterior [%]  N= 154  mean (SD) | 28  -19.9 (4.8) | 59  -19.5 (4.2) | 34  -18.0 (4.9) | 33  -20.9 (5.3) | 0.098 |
| Mid anterior [%]  N= 172  mean (SD) | 33  -17.3 (3.0) | 65  -18.1 (2.6) | 38  -18.0 (3.2) | 36  -16.3 (7.7) | 0.372* |
| Basal anterior [%]  N= 182  mean (SD) | 34  -18.0 (3.0) | 70  -18.1 (2.8) | 40  -18.2 (3.6) | 38  -17.7 (3.7) | 0.883 |
|  |  |  |  |  |  |
| LPS 3C [%]  N= 165  mean (SD) | 26  -17.5 (1.7) | 62  -18.5 (2.7) | 39  -18.9 (2.9) | 38  -18.2 (3.3) | 0.190 |
| Basal posterior [%]  N= 180  mean (SD) | 32  -17.1 (2.9) | 69  -18.5 (3.1) | 39  -18.6 (3.4) | 40  -17.1 (8.4) | 0.086  * |
| Mid posterior [%]  N= 180  mean (SD) | 33  -17.3 (2.1) | 68  -18.7 (3.0) | 40  -17.9 (3.9) | 39  -18.4 (3.7) | 0.079* |
| Apical posterior [%]  N= 166  mean (SD) | 30  -17.8 (3.5) | 63  -19.1 (4.8) | 35  -18.8 (4.8) | 38  -18.8 (4.2) | 0.662 |
| Apical anteroseptal [%]  N= 167  mean (SD) | 30  -19.8 (3.8) | 64  -19.6 (7.3) | 39  -21.2 (4.7) | 34  -20.5 (5.1) | 0.546 |
| Mid anteroseptal [%]  N= 184  mean (SD) | 34  -18.9 (2.5) | 70  -18.5 (3.2) | 40  -19.5 (3.7) | 40  -18.2 (4.1) | 0.338 |
| Basal anteroseptal [%]  N= 185  mean (SD) | 35  -16.7 (2.4) | 70  -16.0 (3.2) | 40  -16.6 (3.8) | 40  -15.6 (3.5) | 0.304* |

Values are given as mean±standard deviation (ANOVA or *Welch´s test depending on equality of variance in Levene´s test)**;** LPS = longitudinal peak systolic strain. To increase statistical power, the highest two decades were combined.

4C = four chamber view; 3C = three chamber view; 2C = two chamber view.

**Table B:** Left ventricular global peak systolic longitudinal strain and – indented – the respective segmental longitudinal peak systolic strain values in female participants by age group

| Age group (years) | 30-39 | 40-49 | 50-59 | 60-79 | p |
| --- | --- | --- | --- | --- | --- |
|  |  |  |  |  |  |
| GL-PSS [%]  N= 177 (%)  mean (SD) | 28 (16)  -20.7 (2.2) | 73 (41)  -20.5 (1.8) | 42 (24)  -20.7 (1.6) | 34 (19)  -19.6 (2.4) | 0.120* |
|  |  |  |  |  |  |
| LPS 4C [%]  N= 208  mean (SD) | 37  -19.9 (2.3) | 87  -20.2 (2.4) | 46  -20.3 (2.3) | 38  -19.4 (2.5) | 0.300 |
| Basal septal [%]  N= 249  mean (SD) | 48  -18.1 (2.9) | 106  -17.8 (2.7) | 55  -16.2 (3.5) | 40  -15.7 (3.5) | 0.0001* |
| Mid septal [%]  N= 250  mean (SD) | 48  -20.3 (2.3) | 106  -20.4 (2.3) | 55  -19.0 (3.0) | 41  -18.4 (2.9) | 0.0001 |
| Apical septal [%]  N= 243  mean (SD) | 46  -22.6 (4.1) | 104  -23.3 (4.0) | 54  -23.9 (4.0) | 39  -23.0 (4.2) | 0.423 |
| Apical lateral [%]  N= 223  mean (SD) | 45  -20.6 (4.5) | 89  -20.4 (4.9) | 49  -21.8 (4.2) | 40  -19.4 (6.0) | 0.122 |
| Mid lateral [%]  N= 242  mean (SD) | 47  -19.9 (2.9) | 99  -19.3 (3.5) | 55  -19.6 (3.8) | 41  -18.4 (4.2) | 0.240 |
| Basal lateral [%]  N= 243  mean (SD) | 44  -18.9 (4.3) | 104  -19.1 (5.7) | 54  -19.9 (3.6) | 41  -20.0 (3.8) | 0.494 |
|  |  |  |  |  |  |
| LPS 2C [%]  N= 194  mean (SD) | 34  -21.5 (2.9) | 81  -21.5 (2.5) | 44  -21.5 (1.7) | 35  -20.2 (2.7) | 0.087* |
| Basal inferior [%]  N= 239  mean (SD) | 45  -21.9 (3.9) | 101  -21.0 (3.3) | 53  -21.0 (4.1) | 40  -19.6 (3.6) | 0.041 |
| Mid inferior [%]  N= 237  mean (SD) | 43  -22.1 (3.5) | 101  -21.8 (3.0) | 53  -21.5 (3.1) | 40  -20.7 (2.6) | 0.164 |
| Apical inferior [%]  N= 228  mean (SD) | 43  -24.6 (4.9) | 96  -24.5 (4.3) | 51  -25.3 (4.0) | 38  -23.8 (3.9) | 0.383 |
| Apical anterior [%]  N= 198  mean (SD) | 38  -20.2 (5.3) | 81  -21.3 (5.4) | 44  -22.1 (4.6) | 35  -19.3 (5.6) | 0.081 |
| Mid anterior [%]  N= 210  mean (SD) | 36  -20.4 (3.5) | 86  -20.1 (3.8) | 51  -19.7 (3.3) | 37  -18.2 (4.5) | 0.054 |
| Basal anterior [%]  N= 231  mean (SD) | 43  -21.2 (3.9) | 95  -20.3 (4.0) | 53  -19.9 (3.7) | 40  -19.1 (4.1) | 0.091 |
|  |  |  |  |  |  |
| LPS 3C [%]  N=195  mean (SD) | 32  -20.6 (2.5) | 82  -20.0 (2.3) | 44  -20.2 (2.5) | 37  -19.1 (3.1) | 0.088 |
| Basal posterior [%]  N= 225  mean (SD) | 39  -19.7 (3.9) | 93  -20.4 (3.5) | 54  -19.0 (4.4) | 39  -19.2 (7.4) | 0.266* |
| Mid posterior [%]  N= 229  mean (SD) | 41  -20.0 (3.1) | 94  -19.9 (3.1) | 54  -18.8 (4.0) | 40  -18.6 (4.6) | 0.139* |
| Apical posterior [%]  N= 209  mean (SD) | 35  -21.1 (4.7) | 83  -20.2 (5.0) | 52  -21.2 (3.8) | 39  -19.2 (5.0) | 0.169 |
| Apical anteroseptal [%]  N= 205  mean (SD) | 35  -22.8 (4.9) | 87  -22.0 (5.2) | 50  -24.3 (4.2) | 34  -22.3 (3.5) | 0.041 |
| Mid anteroseptal [%]  N= 228  mean (SD) | 41  -20.8 (3.4) | 93  -20.1 (3.1) | 53  -20.3 (3.1) | 41  -18.6 (3.8) | 0.018 |
| Basal anteroseptal [%]  N= 230  mean (SD) | 41  -18.5 (3.2) | 94  -17.9 (3.5) | 54  -16.8 (3.2) | 41  -16.0 (3.6) | 0.002 |

Values are given as mean±standard deviation (ANOVA or *Welch´s test depending on equality of variance in Levene´s test)**;** LPS = longitudinal peak systolic strain. To increase statistical power, the highest two decades were combined.

4C = four chamber view; 3C = three chamber view; 2C = two chamber view.

**Table C:** Impact of cardiovascular risk factors on global longitudinal peak systolic strain (GL-PSS) in the total cohort and according to sex.

|  | **Δ GL-PSS (total)** | **Δ GL-PSS (males)** | **Δ GL-PSS (females)** | **P for interaction** (males/females) |
| --- | --- | --- | --- | --- |
| **Hypertension** | +0.3  [-0.0; +0.6] | -0.1  [-0.5; +0.2] | +0.7  [+0.3; +1.1]** | 0.004 |
| **Dyslipidemia** | +0.8  [+0.4; +1.2]*** | +0.3  [-0.2; +0.8] | +1.2  [+0.6; +1.8]*** | 0.03 |
| **Obesity** | +0.7  [+0.3; +1.1]*** | +0.7  [+0.2; +1.2]** | +0.9  [+0.3; +1.4]** | 0.69 |
| **Diabetes** | +0.5  [-0.0; +1.1] | +0.1  [-0.5; +0.9] | +0.7  [-0.1; +1.6] | 0.27 |
| **Smoking** | +0.2  [-0.1; +0.4] | +0.3  [-0.1; +0.6] | -0.2  [-0.5; +0.2] | 0.10 |

We show the delta (ƌ) GL-PSS (regression coefficient, n=1218) caused by the respective risk factor indicating the change in GL-PSS associated with the presence of the respective risk factor. *p<0.05; **p<0.01, ***p<0.001.

Hypertension = blood pressure ≥140/90 mmHg, dyslipidemia = low density lipoprotein ≥190 mg/dl, obesity = body mass index >30 kg/m^2^, diabetes mellitus = diabetic medication, HbA1c >6.5%, fasting plasma glucose >7.0 mmol/l or 2h-plasma glucose >11.1 mmol/l, smoking = current or ex-smoker.

**Table D:** Impact of cardiovascular risk factors on systolic strain rate in the total cohort and according to sex.

|  | **Δ GL-SSR (total)** | **Δ GL-SSR (males)** | **Δ GL-SSR (females)** | **P for interaction** (males/females) | | |
| --- | --- | --- | --- | --- | --- | --- |
| **Hypertension** | +0.03  [+0.01; +0.04]** | +0.01  [-0.02; +0.03] | +0.04  [+0.02; +0.07]*** | | 0.02 |  |
| **Dyslipidemia** | +0.03  [+0.00; +0.05]* | +0.01  [-0.02; +0.04] | +0.05  [+0.01; +0.08]** | | 0.07 |  |
| **Obesity** | +0.04  [+0.02; +0.06]*** | +0.02  [-0.01; +0.05] | +0.06  [+0.03; +0.09]*** | | 0.047 |  |
| **Diabetes** | +0.01  [-0.02; +0.04] | -0.01  [-0.04; +0.03] | +0.03  [-0.02; +0.08] | | 0.32 |  |
| **Smoking** | +0.01  [-0.00; +0.03] | +0.00  [-0.02; +0.02] | +0.02  [-0.00; +0.04] | | 0.35 |  |

We show the delta (ƌ) systolic strain rate (regression coefficient, n=1506) caused by the respective risk factor indicating the change in systolic strain rate associated with the presence of the respective risk factor. *p<0.05; **p<0.01, ***p<0.001.

Hypertension = blood pressure ≥140/90 mmHg, dyslipidemia = low density lipoprotein ≥190 mg/dl, obesity = body mass index >30 kg/m^2^, diabetes mellitus = diabetic medication, HbA1c >6.5%, fasting plasma glucose >7.0 mmol/l or 2h-plasma glucose >11.1 mmol/l, smoking = current or ex-smoker.

**Table E:** Impact of cardiovascular risk factors on early diastolic strain rate in the total cohort and according to sex.

|  | **Δ GL-EDSR (total)** | **Δ GL-EDSR (males)** | **Δ GL-EDSR (females)** | | **P for interaction** (males/females) |
| --- | --- | --- | --- | --- | --- |
| **Hypertension** | -0.18  [-0.22; -0.14]*** | -0.10  [-0.15; -0.05]*** | -0.24  [-0.29; -0.19]*** | <0.001 | |
| **Dyslipidemia** | -0.12  [-0.17; -0.06]*** | -0.02  [-0.09; +0.04] | -0.19  [-0.27; -0.12]*** | 0.001 | |
| **Obesity** | -0.12  [-0.18; -0.07]*** | -0.12  [-0.19; -0.06]*** | -0.14  [-0.21; -0.07]*** | 0.72 | |
| **Diabetes** | -0.04  [-0.11; +0.04] | +0.04  [-0.05; +0.12] | -0.08  [-0.19; +0.04] | 0.11 | |
| **Smoking** | -0.02  [-0.06; +0.01] | -0.01  [-0.06; +0.04] | +0.00  [-0.05; +0.05] | 0.73 | |

We show the delta (ƌ) early diastolic strain rate (regression coefficient, n=1506) caused by the respective risk factor indicating the change in early diastolic strain rate associated with the presence of the respective risk factor. *p<0.05; **p<0.01, ***p<0.001.

Hypertension = blood pressure ≥140/90 mmHg, dyslipidemia = low density lipoprotein ≥190 mg/dl, obesity = body mass index >30 kg/m^2^, diabetes mellitus = diabetic medication, HbA1c >6.5%, fasting plasma glucose >7.0 mmol/l or 2h-plasma glucose >11.1 mmol/l, smoking = current or ex-smoker.

**Table F:** Impact of cardiovascular risk factors on late diastolic strain rate in the total cohort and according to sex.

|  | **Δ GL-LDSR (total)** | **Δ GL-LDSR (males)** | **Δ GL-LDSR (females)** | | **P for interaction** (males/females) | |
| --- | --- | --- | --- | --- | --- | --- |
| **Hypertension** | +0.09  [+0.06; +0.11]*** | +0.06  [+0.03; +0.09]*** | +0.11  [+0.08; +0.14]*** | 0.02 | |  |
| **Dyslipidemia** | +0.02  [-0.01; +0.06] | +0.00  [-0.04; +0.04] | +0.05  [+0.00; +0.10]* | 0.13 | |  |
| **Obesity** | -0.00  [-0.03; +0.03] | -0.02  [-0.06; +0.02] | +0.02  [-0.02; +0.06] | 0.16 | |  |
| **Diabetes** | -0.00  [-0.05; +0.04] | -0.02  [-0.07; +0.03] | +0.02  [-0.04; +0.09] | 0.28 | |  |
| **Smoking** | -0.01  [-0.03; +0.01] | +0.01  [-0.02; +0.04] | -0.02  [-0.05; +0.01] | 0.18 | |  |

We show the delta (ƌ) late diastolic strain rate (regression coefficient, n=1500) caused by the respective risk factor indicating the change in late diastolic strain rate associated with the presence of the respective risk factor. *p<0.05; **p<0.01, ***p<0.001.

Hypertension = blood pressure ≥140/90 mmHg, dyslipidemia = low density lipoprotein ≥190 mg/dl, obesity = body mass index >30 kg/m^2^, diabetes mellitus = diabetic medication, HbA1c >6.5%, fasting plasma glucose >7.0 mmol/l or 2h-plasma glucose >11.1 mmol/l, smoking = current or ex-smoker.

Supplementary figures

**
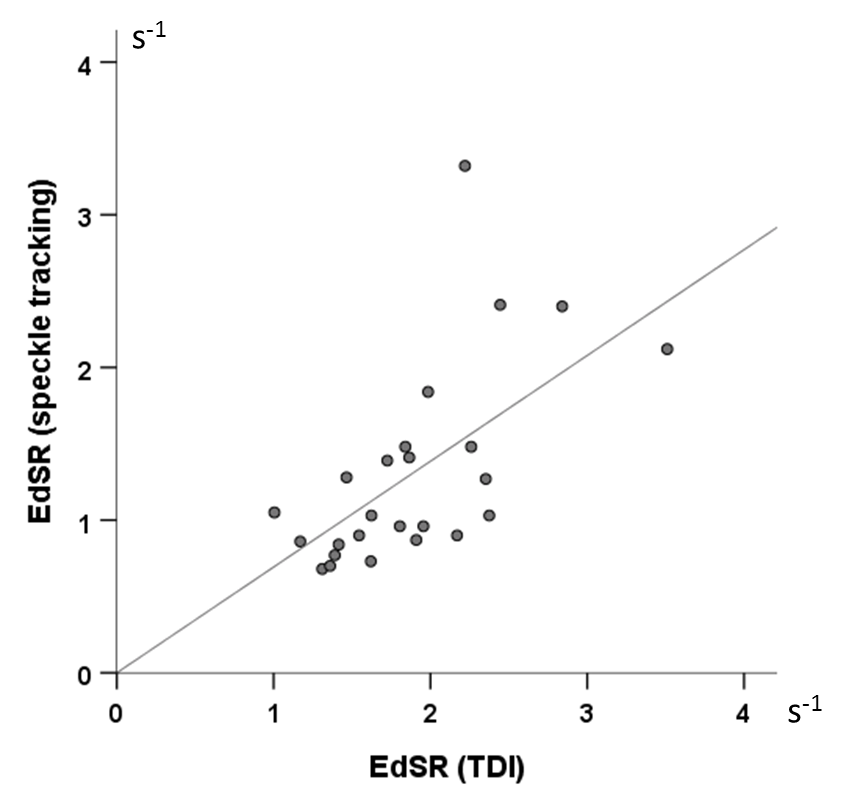
**

**Figure A: Correlation of speckle tracking derived global early diastolic strain rate (EdSR) and tissue Doppler imaging (TDI) derived early diastolic strain rate (average of mid-septal and mid-lateral wall measurement).**


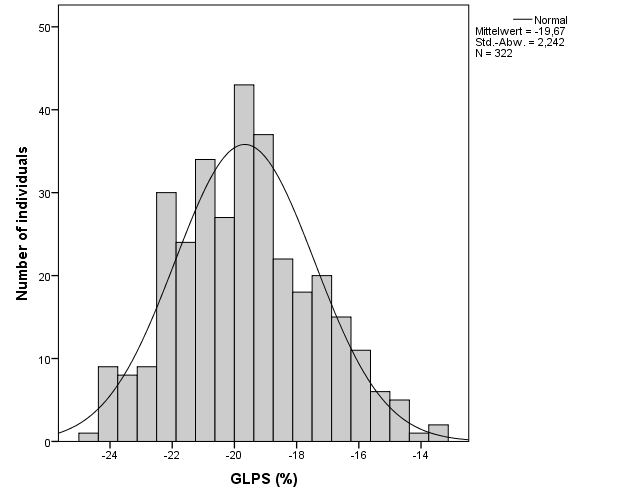

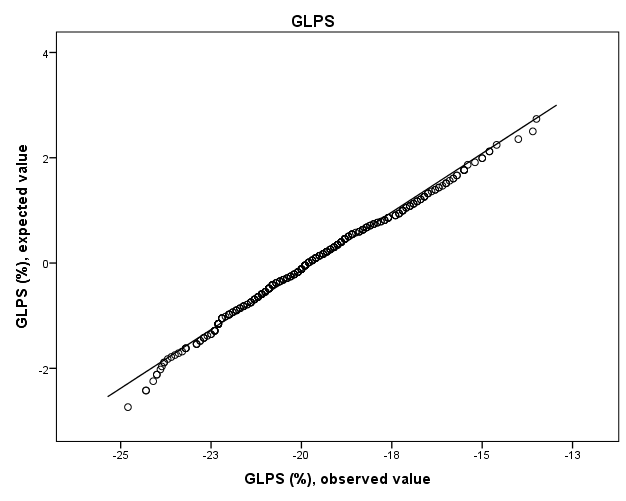


**Figure B:** a) Histogram and b) Q-Q plot (standard normal distribution) of combined averaged global longitudinal peak systolic strain (GL-PSS) in 323 individuals without cardiovascular risk factors (mean age 49±11 years, 55% females). The test for normal distribution was carried out to decide if between group differences or rather between groups respective ratios should be modelled. The approximate normal distribution supports an additive nature of this quantity and hence modeling of differences in justified. Furthermore, normality allows computing 95% reference ranges as mean +/-1.96 SD


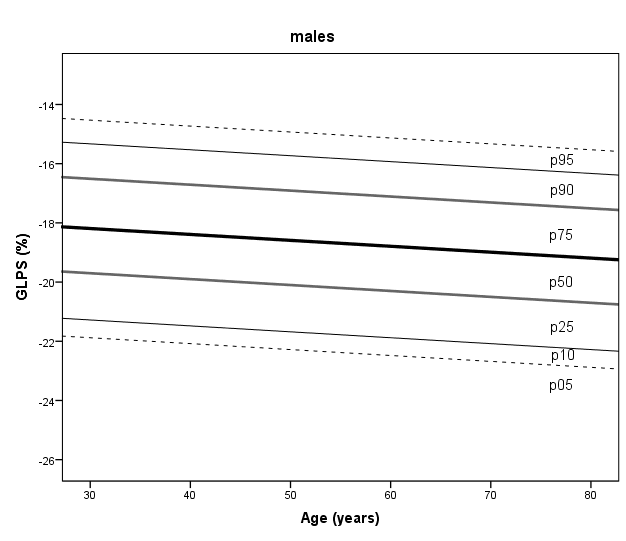


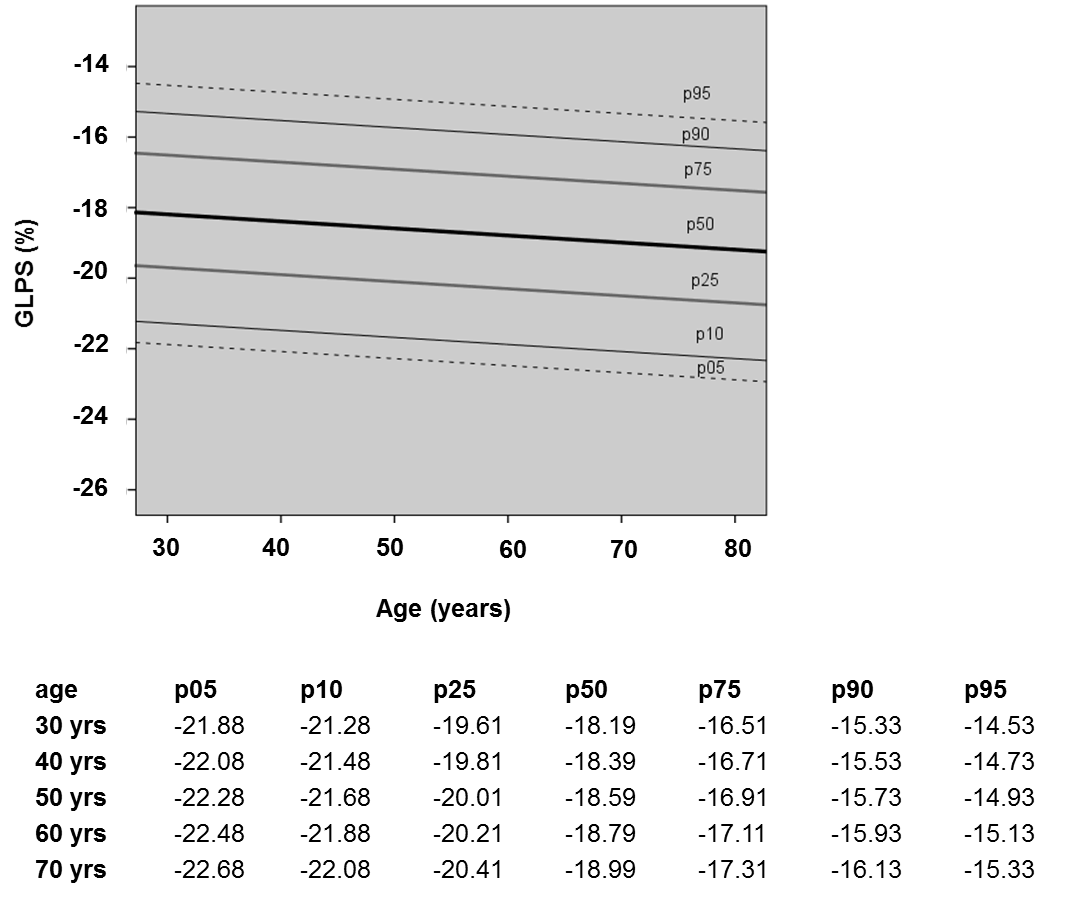


**Figure C:** Percentiles (p) of global longitudinal peak systolic strain (GL-PSS) in men (n=146, mean age 49±11years)


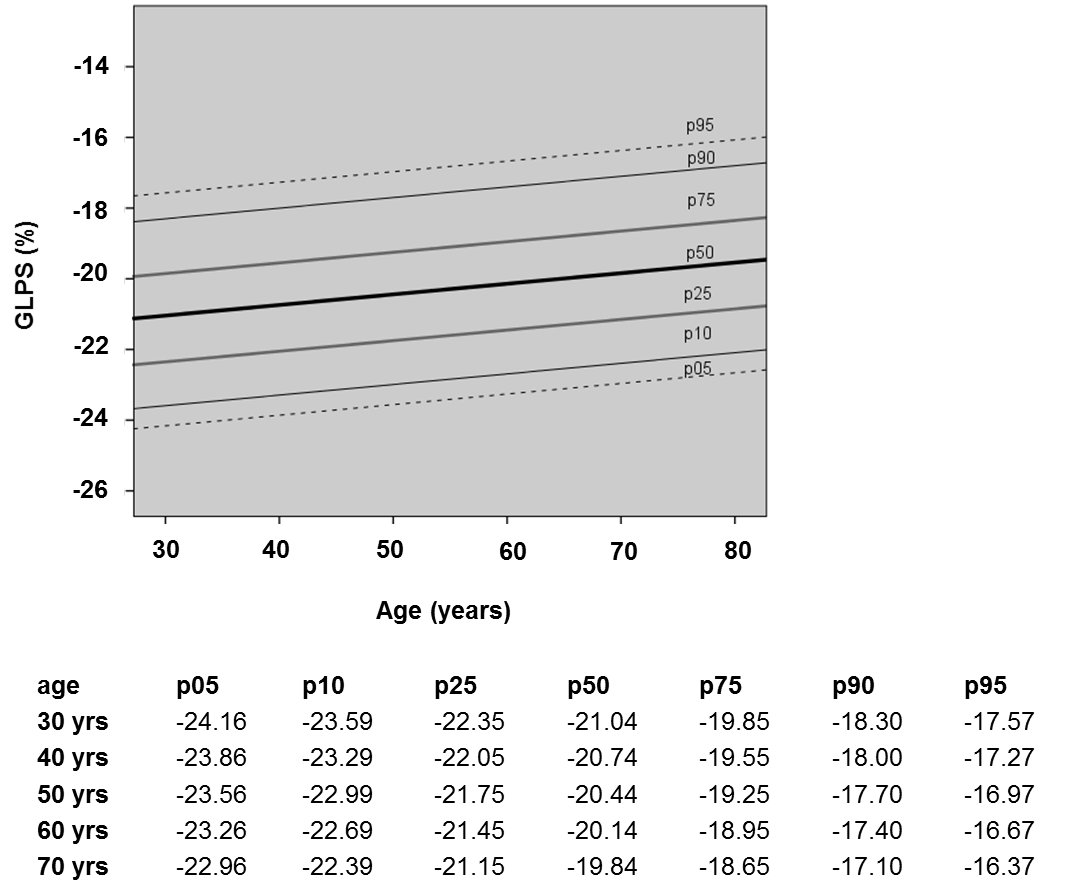

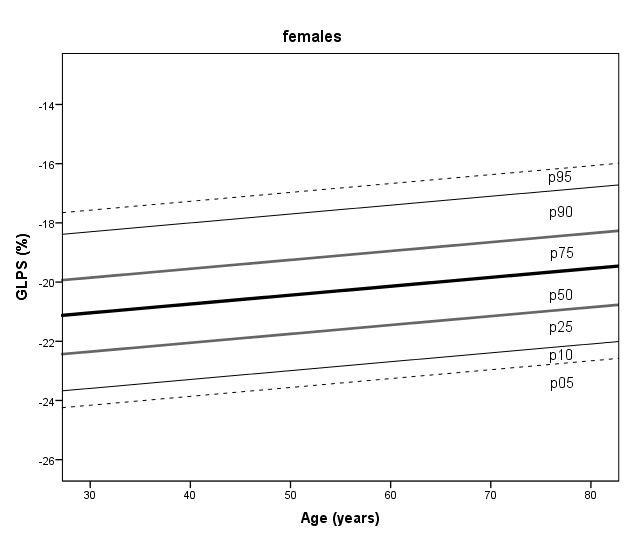


**Figure D:** Percentiles (p) of global longitudinal peak systolic strain (GL-PSS) in women (n=177, mean age 49±10 years).


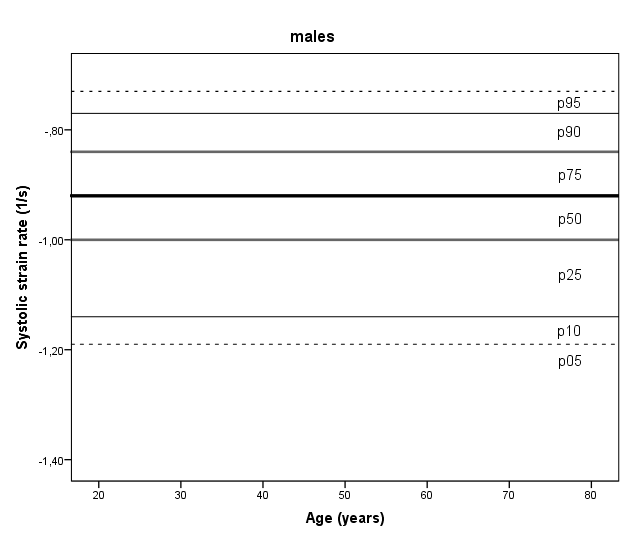


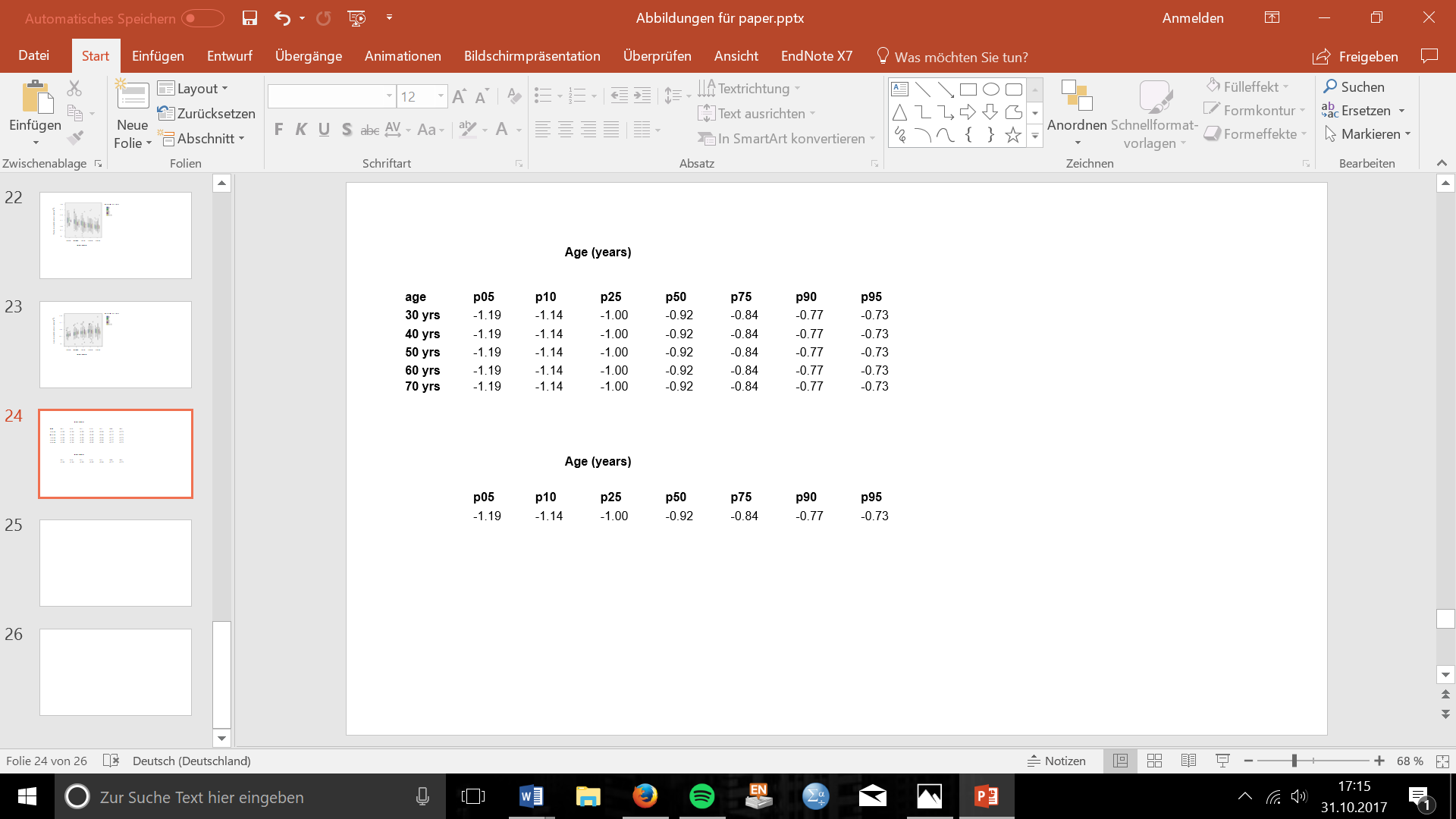


**Figure E:** Percentiles (p) of systolic strain rate in men (n=182, 49±12 years)


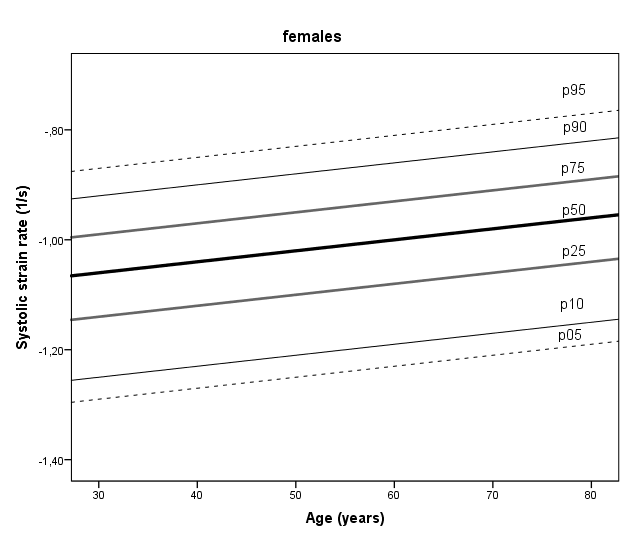

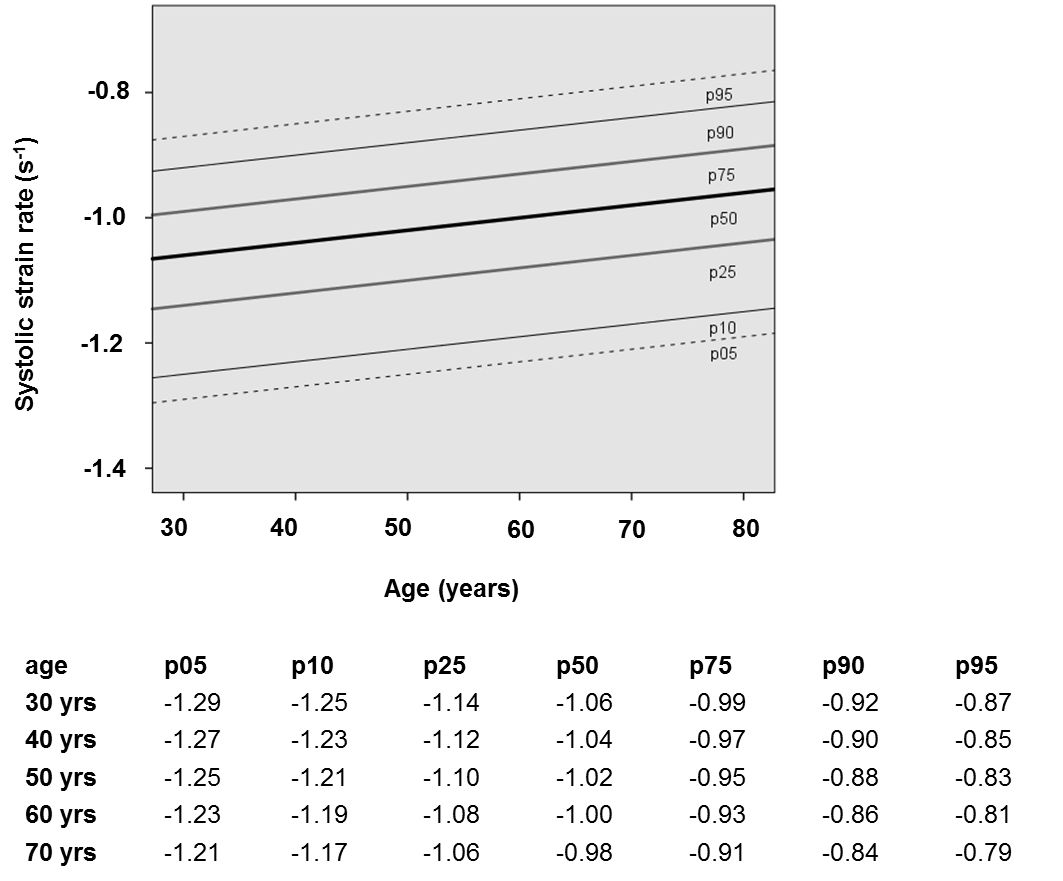


**Figure F:** Percentiles (p) of systolic strain rate in women (n=228, mean age 49±10 years)


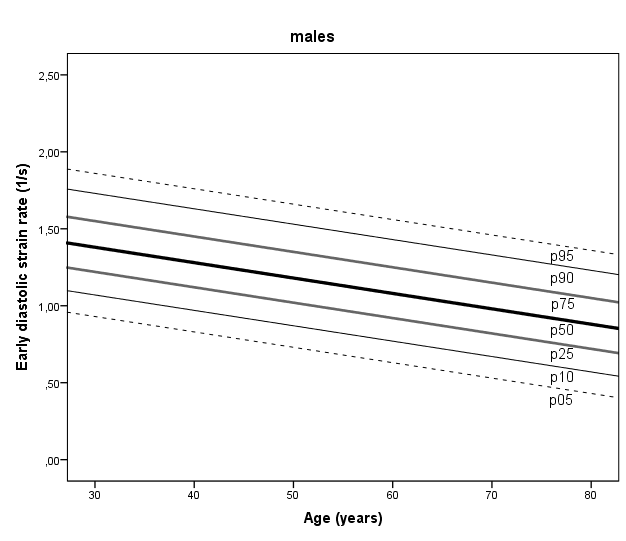


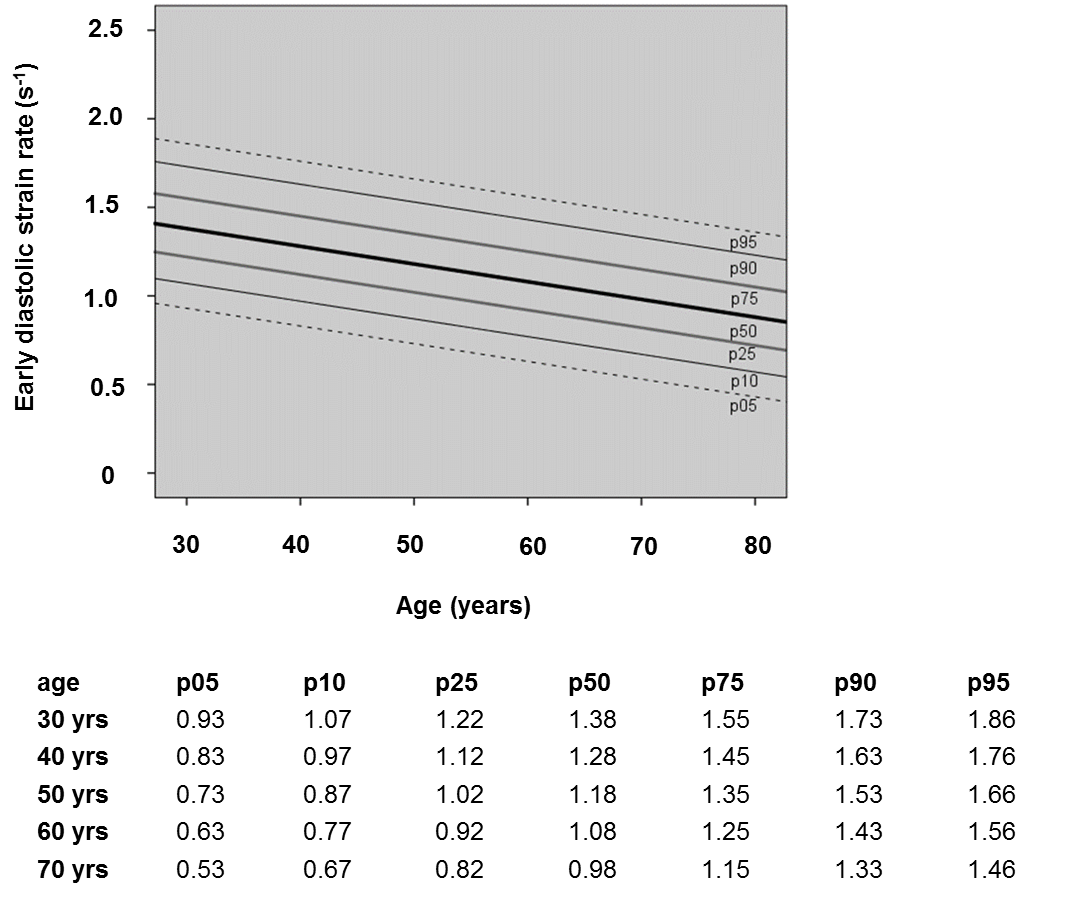


**Figure G:** Percentiles (p) of early diastolic strain rate in men (n=183, mean age 49±12 years)


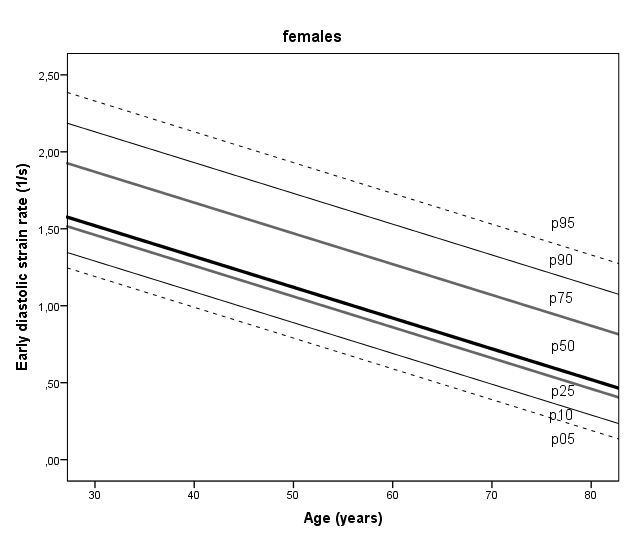


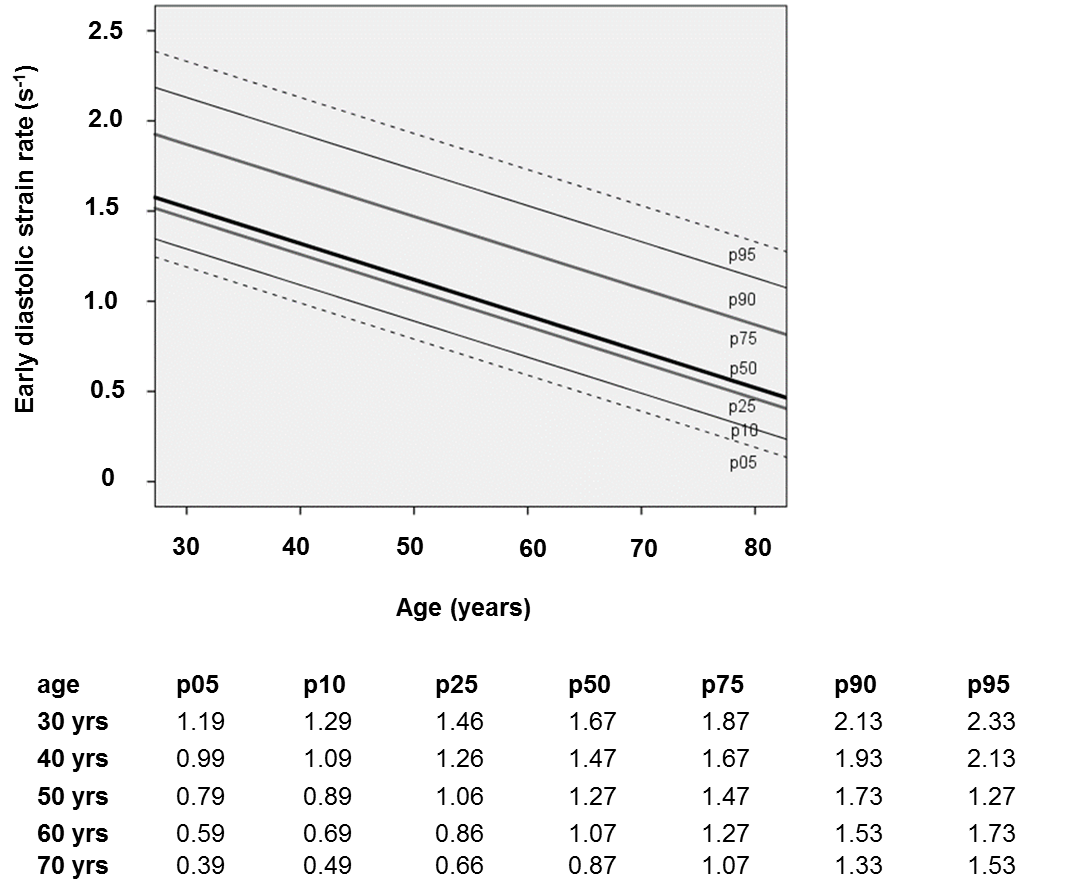


**Figure H:** Percentiles (p) of early diastolic strain rate in women (n=228, mean age 49±10 years)


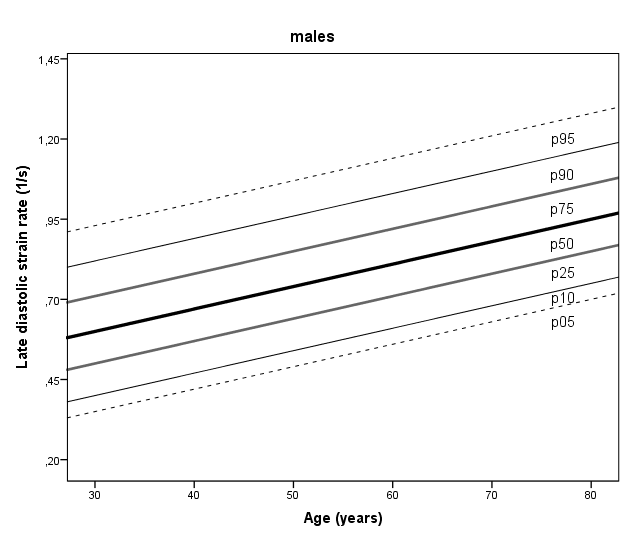


**
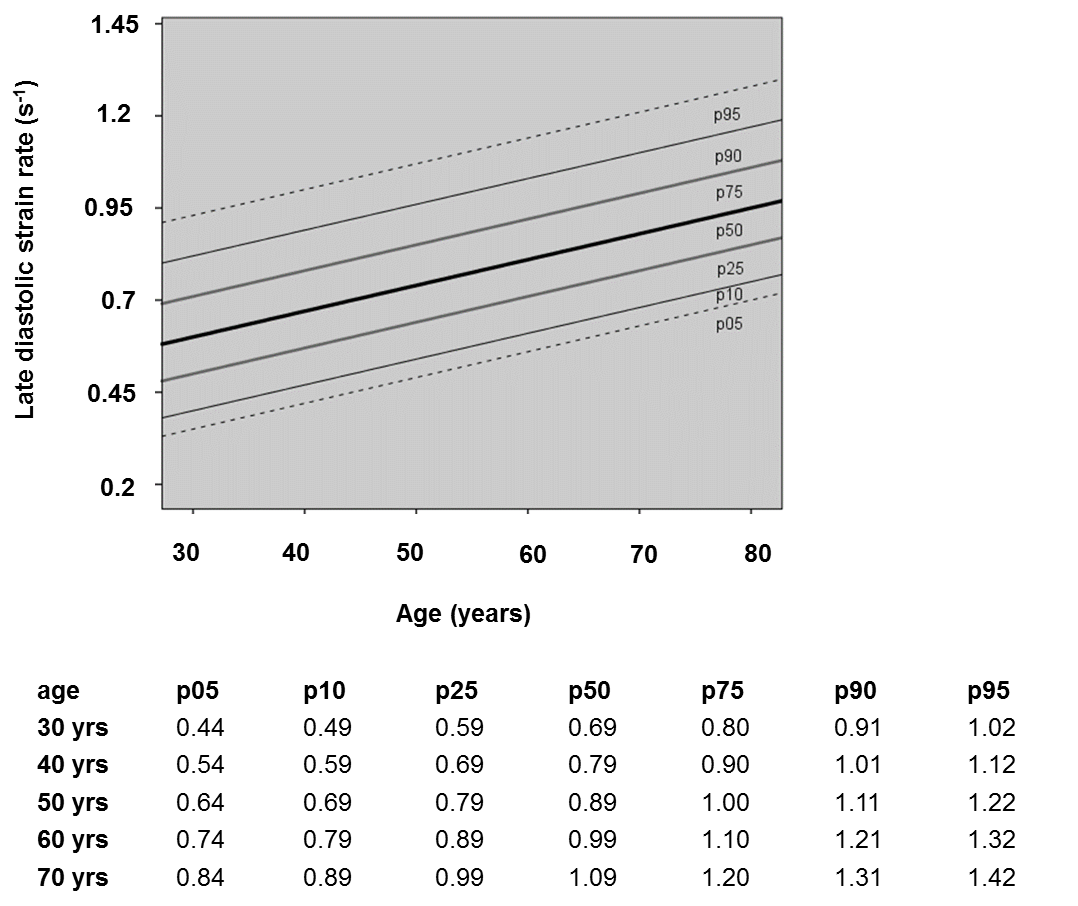
**

**Figure I:** Percentiles (p) of late diastolic strain rate in men (n=182, mean age 49±12 years)


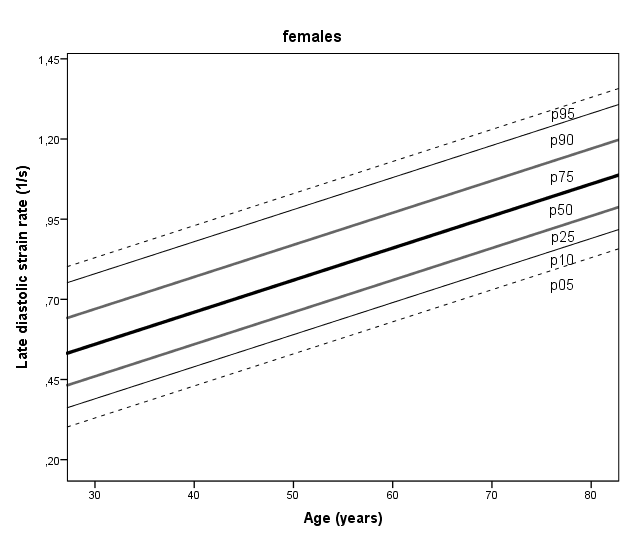


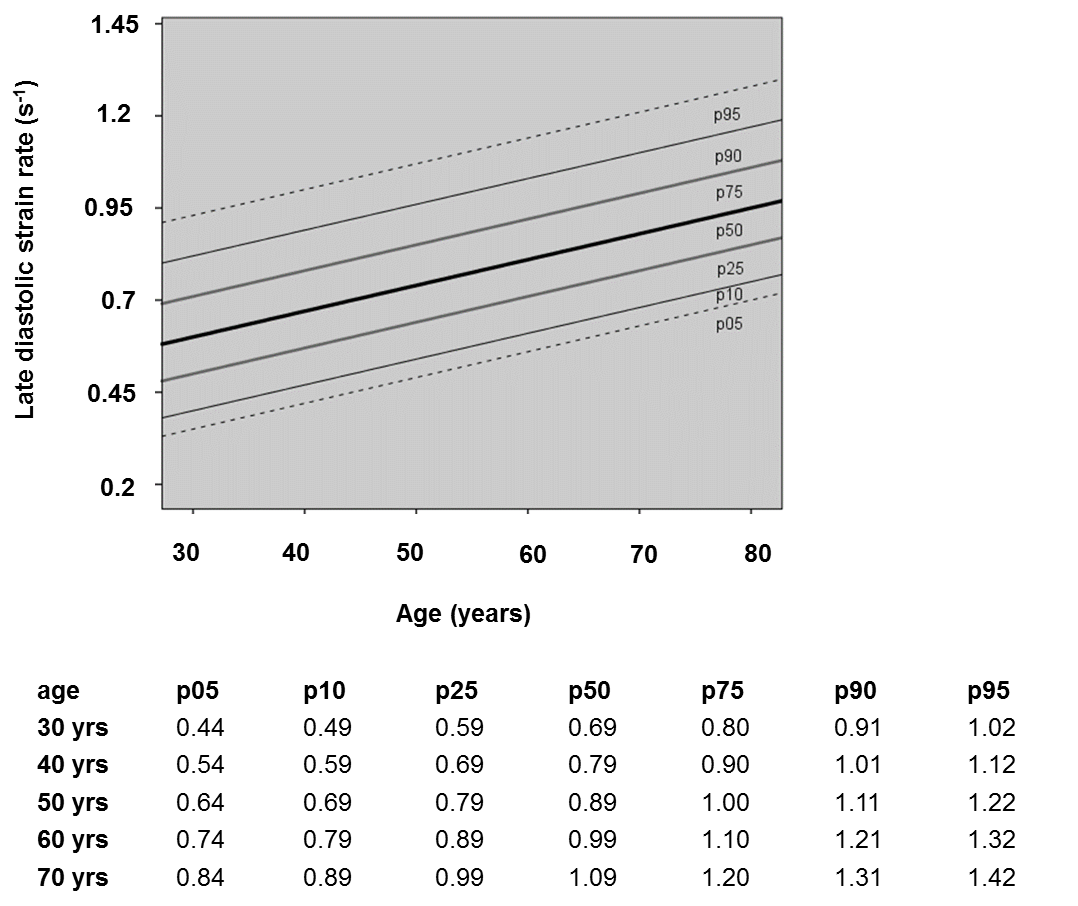


**Figure J:** Percentiles (p) of late diastolic strain rate in women (n=226, mean age 49±10 years)


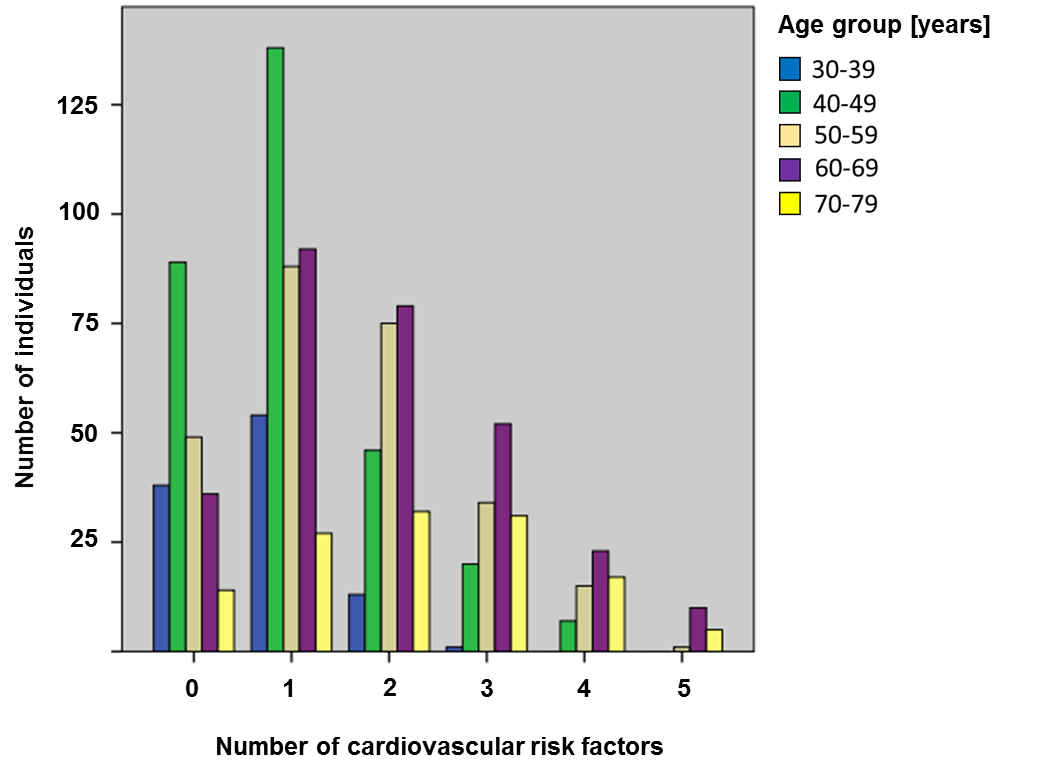


**Figure K:** Number of male individuals with 0-5 cardiovascular risk factors by age decades (n=594).

**
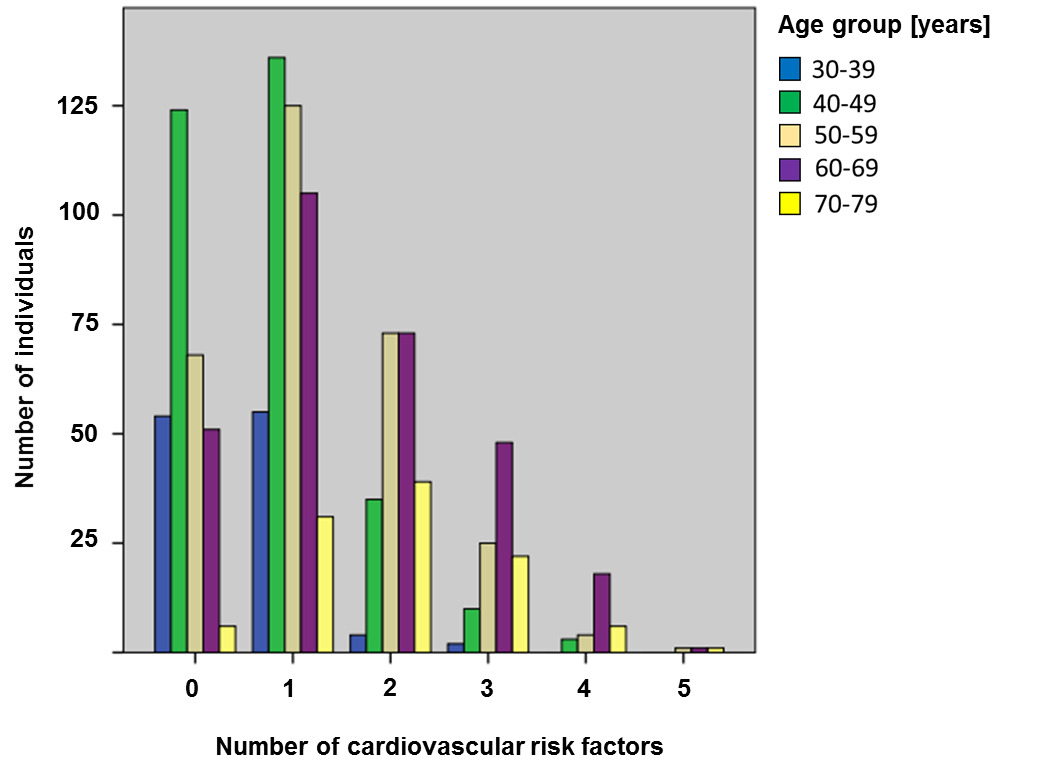
**

**Figure L**: Number of female individuals with 0-5 cardiovascular risk factors by age decades (n=624).
